# A note on Marcinkiewicz integrals supported by submanifolds

**DOI:** 10.1186/s13660-018-1822-8

**Published:** 2018-09-04

**Authors:** Feng Liu

**Affiliations:** College of Mathematics and Systems Sciences, Shandong University of Science and Technology, Qingdao, China

**Keywords:** 42B20, 42B25, 47G10, Polynomial compound mappings, $H^{1}(\mathrm{S}^{n-1})$, Triebel–Lizorkin spaces, Besov spaces

## Abstract

In the present paper, we establish the boundedness and continuity of the parametric Marcinkiewicz integrals with rough kernels associated to polynomial mapping $\mathcal{P}$ as well as the corresponding compound submanifolds, which is defined by

$$ \mathcal{M}_{h,\Omega ,\mathcal{P}}^{\rho }f(x)= \biggl( \int_{0}^{\infty } \biggl\vert \frac{1}{t^{\rho }} \int_{ \vert y \vert \leq t}\frac{\Omega (y)h( \vert y \vert )}{ \vert y \vert ^{n- \rho }}f \bigl(x-\mathcal{P}(y) \bigr)\,dy \biggr\vert ^{2}\frac{dt}{t} \biggr)^{1/2}, $$

on the Triebel–Lizorkin spaces and Besov spaces when $\Omega \in H ^{1}(\mathrm{S}^{n-1})$ and $h\in \Delta_{\gamma }(\mathbb{R}_{+})$ for some $\gamma >1$. Our main results represent significant improvements and natural extensions of what was known previously.

## Introduction

As is well known, the Triebel–Lizorkin spaces and Besov spaces contain many important function spaces, such as Lebesgue spaces, Hardy spaces, Sobolev spaces and so on. During the last several years, a considerable amount of attention has been given to investigate the boundedness for several integral operators on the Triebel–Lizorkin spaces and Besov spaces. For examples, see [1–6] for singular integrals, [7–13] for Marcinkiewicz integrals, [14] for the Littlewood–Paley functions, [15–18] for maximal functions. In this paper we continue to focus on this topic. More precisely, we aim to establish the boundedness and continuity of parametric Marcinkiewicz integral operators associated to polynomial compound mappings with rough kernels in Hardy spaces $H^{1}(\mathrm{S} ^{n-1})$ on the Triebel–Lizorkin spaces and Besov spaces.

We now recall the definitions of Triebel–Lizorkin spaces and Besov spaces.

### Definition 1.1

Let $d\geq 2$ and $\mathcal{S}'( \mathbb{R}^{d})$ be the tempered distribution class on $\mathbb{R} ^{d}$. For $\alpha \in \mathbb{R}$ and $0< p$, $q\le \infty $ ($p\neq\infty$), the homogeneous Triebel–Lizorkin spaces $\dot{F}_{\alpha }^{p,q}(\mathbb{R}^{d})$ and Besov spaces $\dot{B}_{\alpha }^{p,q}( \mathbb{R}^{d})$ are defined by

1.1 $${\dot{F}}_{\alpha }^{p,q}\left(R^{d}\right):=\left\{f\in S^{\prime }\left(R^{d}\right):{∥f∥}_{{\dot{F}}_{\alpha }^{p,q}(R^{d})}={∥{\left(\sum_{i\in Z}2^{-i\alpha q}|\Psi_{i}*f{|}^{q}\right)}^{1/q}∥}_{L^{p}(R^{d})}<\infty \right\};$$

1.2 $${\dot{B}}_{\alpha }^{p,q}\left(R^{d}\right):=\left\{f\in S^{\prime }\left(R^{d}\right):{∥f∥}_{{\dot{B}}_{\alpha }^{p,q}(R^{d})}={\left(\sum_{i\in Z}2^{-i\alpha q}{∥\Psi_{i}*f∥}_{L^{p}(R^{d})}^{q}\right)}^{1/q}<\infty \right\},$$

where $\widehat{\Psi _{i}}(\xi )=\phi (2^{i}\xi )$ for $i\in \mathbb{Z}$ and $\phi \in \mathcal{C}_{c}^{\infty } (\mathbb{R}^{d})$ satisfies the conditions: $0\leq \phi (x)\leq 1$; $\operatorname{supp}(\phi ) \subset \{x: 1/2\leq \vert x \vert \leq 2\}$; $\phi (x)>c>0$ if $3/5\leq \vert x \vert \leq 5/3$. The inhomogeneous versions of Triebel–Lizorkin spaces and Besov spaces, which are denoted by $F_{\alpha }^{p,q} (\mathbb{R}^{d})$ and $B_{\alpha }^{p,q}(\mathbb{R}^{d})$, respectively, are obtained by adding the term ${∥\Theta *f∥}_{L^{p}(R^{d})}$ to the right hand side of (1.1) or (1.2) with $\sum_{i\in \mathbb{Z}}$ replaced by $\sum_{i\geq 1}$, where $\Theta \in \mathcal{S}(\mathbb{R}^{d})$ (the Schwartz class), $\operatorname{supp}(\hat{\Theta })\subset \{\xi : \vert \xi \vert \leq 2\}$, $\hat{\Theta }(x)>c>0$ if $\vert x \vert \leq 5/3$.

The following properties of the above spaces are well known (see [19–21] for more details):

1.3 $$\begin{aligned}& \dot{F}_{0}^{p,2} \bigl(\mathbb{R}^{d} \bigr)=L^{p} \bigl(\mathbb{R}^{d} \bigr) \quad \mbox{for } 1< p< \infty ; \end{aligned}$$

1.4 $$\begin{aligned}& \dot{F}_{\alpha }^{p,p} \bigl(\mathbb{R}^{d} \bigr)= \dot{B}_{\alpha }^{p,p} \bigl( \mathbb{R}^{d} \bigr)\quad \mbox{for } \alpha \in \mathbb{R} \mbox{ and } 1< p< \infty ; \end{aligned}$$

1.5 $$\begin{array}{rl} & F_{\alpha }^{p,q}\left(R^{d}\right)∼{\dot{F}}_{\alpha }^{p,q}\left(R^{d}\right)\cap L^{p}\left(R^{d}\right)\;\text{and}\;\\ & {∥f∥}_{F_{\alpha }^{p,q}(R^{d})}∼{∥f∥}_{{\dot{F}}_{\alpha }^{p,q}(R^{d})}+{∥f∥}_{L^{p}(R^{d})}\;\text{for }\alpha >0; \end{array}$$

1.6 $$\begin{array}{rl} & B_{\alpha }^{p,q}\left(R^{d}\right)∼{\dot{B}}_{\alpha }^{p,q}\left(R^{d}\right)\cap L^{p}\left(R^{d}\right)\;\text{and}\\ & {∥f∥}_{B_{\alpha }^{p,q}(R^{d})}∼{∥f∥}_{{\dot{B}}_{\alpha }^{p,q}(R^{d})}+{∥f∥}_{L^{p}(R^{d})}\;\text{for }\alpha >0. \end{array}$$

Let $n\geq 2$ and $\mathrm{S}^{n-1}$ be the unit sphere in $\mathbb{R} ^{n}$ equipped with the normalized Lebesgue measure *dσ*. Assume that $\Omega \in L^{1}(\mathrm{S}^{n-1})$ is a function of homogeneous of degree zero and satisfies the cancelation condition

1.7 $$ \int_{\mathrm{S}^{n-1}}\Omega (u)\,d\sigma (u)=0. $$

We denote by $\Delta_{\gamma }(\mathbb{R}_{+})$ ($\gamma \geq 1$) the set of all measurable functions *h* defined on $\mathbb{R}_{+}:=(0,\infty )$ satisfying

$${∥h∥}_{\Delta_{\gamma }(R_{+})}:=\underset{R>0}{\sup}{\left(R^{-1}\int_{0}^{R}|h(t){|}^{\gamma }\;dt\right)}^{1/\gamma }<\infty .$$

In 1986, Stein [22] first introduced the singular Radon transforms $T_{h,\Omega ,\mathcal{P}}$ by

1.8 $$ T_{h,\Omega ,\mathcal{P}}f(x)=\mathrm{p.v.} \int_{\mathbb{R}^{n}}f \bigl(x- \mathcal{P}(y) \bigr)\frac{\Omega (y)h( \vert y \vert )}{ \vert y \vert ^{n}} \,dy. $$

where $\mathcal{P}=(P_{1},P_{2},\ldots ,P_{d})$ is a polynomial mapping from $\mathbb{R}^{n}$ into $\mathbb{R}^{d}$ and $h\in \Delta_{1}( \mathbb{R}_{+})$. Later on, the bounds of $T_{h,\Omega ,\mathcal{P}}$ on $L^{p}$ spaces and other function spaces have been studied by a large number of scholars (see [4, 23, 24] for example). In particular, Chen et al. [4] established the bounds for $T_{h, \Omega , \mathcal{P}}$ on Triebel–Lizorkin spaces and Besov spaces under the condition that $\Omega \in H^{1} (\mathrm{S}^{n-1})$ and $h\in \Delta_{\gamma }(\mathbb{R}_{+})$ for some $\gamma >1$. It should be pointed out that the class of singular Radon transforms $T_{h,\Omega ,\mathcal{P}}$ is closely related to the class of Marcinkiewicz integral operators

1.9 $$ \mathcal{M}_{h,\Omega ,\mathcal{P}}^{\rho }f(x)= \biggl( \int_{0}^{\infty } \biggl\vert \frac{1}{t^{\rho }} \int_{ \vert y \vert \leq t}\frac{\Omega (y)h( \vert y \vert )}{ \vert y \vert ^{n- \rho }}f \bigl(x-\mathcal{P}(y) \bigr)\,dy \biggr\vert ^{2}\frac{dt}{t} \biggr)^{1/2}, $$

where *h*, Ω, $\mathcal{P}$ are given as in (1.8) and $\rho =\sigma +i\tau $ ($\sigma ,\tau \in \mathbb{R}$ and $\sigma >0$). The operators defined in (1.9) have their roots in the classical Marcinkiewicz integral operator $\mathcal{M}_{\Omega }$, which corresponds to the case $\rho =1$, $h(t)\equiv 1$, $n=d$ and $\mathcal{P}(y)=y$. In their fundamental work on the theory of Marcinkiewicz integrals, Stein [25] proved that $\mathcal{M} _{\Omega }$ is of type $(p,p)$ for $1< p\leq 2$ and of weak type $(1,1)$ if $\Omega \in \operatorname{Lip}_{\alpha }(\mathrm{S}^{n-1})$ ($0<\alpha\leq 1$). Subsequently, the study of $\mathcal{M}_{\Omega }$ and its extensions has attracted the attention of many authors. In 2002, Ding et al. [26] observed that $\mathcal{M}_{h,\Omega , \mathcal{P}}^{\rho }$ with $\rho =1$ is bounded on $L^{p}(\mathbb{R} ^{d})$ for $1< p<\infty $ if $\Omega \in H^{1}(\mathrm{S}^{n-1})$ and $h\in L^{\infty }(\mathbb{R}_{+})$. In 2009, Al-Qassem and Pan [27] proved that $\mathcal{M}_{h,\Omega ,\mathcal{P}}^{\rho }$ is of type $(p,p)$ for $\vert 1/p-1/2 \vert <\min \{1/2,1/\gamma '\}$ if $\Omega \in L(\log^{+}L)^{1/2}(\mathrm{S}^{n-1})$ and $h\in \Delta_{ \gamma }(\mathbb{R}_{+})$ for some $\gamma >1$. It is well known that $L(\log^{+}L)^{1/2}(\mathrm{S}^{n-1})$ and $H^{1}(\mathrm{S}^{n-1})$ do not contain each other. We also note that $L^{\infty }(\mathbb{R}_{+})= \Delta_{\infty } (\mathbb{R}_{+})$ and $\Delta_{\gamma_{2}}( \mathbb{R}_{+}) \subsetneq \Delta_{\gamma_{1}}(\mathbb{R}_{+})$ for $\gamma_{2}>\gamma_{1}>0$.

On the other hand, the investigation on the boundedness of Marcinkiewicz integral operators on Triebel–Lizorkin spaces and Besov spaces has attracted the attention of many authors. In 2009, Zhang and Chen [12] observed that $\mathcal{M}_{h,\Omega }^{\rho }$ is bounded on $F_{\alpha }^{p,q}(\mathbb{R}^{d})$ for $0<\alpha <1$ and $1< p$, $q<\infty $ if $\rho =1$, $h\equiv 1$ and $\Omega \in H^{1}( \mathrm{S}^{n-1})$. Subsequently, Zhang and Chen [13] further proved that $\mathcal{M}_{h,\Omega }^{\rho }$ is bounded on $F_{\alpha }^{p,q}(\mathbb{R}^{d})$ for $0<\alpha <1$ and $1+ \frac{n+1}{n+2-1/r}< p$, $q<2+\frac{1-1/r}{n+1}$ if $\rho =1$, $h\in L^{\infty }(\mathbb{R}_{+})$ and $\Omega \in L^{r}(\mathrm{S}^{n-1})$ with $r>1$. Recently, Yabuta [10] improved and extended the above results to the case $\Omega \in H^{1}(\mathrm{S}^{n-1})$ and $h\in \Delta_{\gamma }(\mathbb{R}_{+})$ for some $\gamma >1$. For other interesting work on this topic we refer the reader to [1, 7, 8, 28–33].

Based on the above, a natural question, which arises from the above results, is the following.

### Question A

Is the operator $\mathcal{M}_{h, \Omega , \mathcal{P}}^{\rho }$ bounded on Triebel–Lizorkin spaces and Besov spaces under the condition that $\Omega \in H^{1}(\mathrm{S}^{n-1})$ and $h\in \Delta_{\gamma }(\mathbb{R}_{+})$?

Question A is the main motivation for this work. The main purpose of this paper will not only be to address the above question by treating a more general class of operators but also to establish the corresponding continuity of Marcinkiewicz integral operators on Triebel–Lizorkin spaces and Besov spaces. More precisely, let *h*, Ω, *ρ*, $\mathcal{P}$ be given as in (1.9) and $\varphi :\mathbb{R}_{+}\rightarrow \mathbb{R}$ be a suitable function, we define the parametric Marcinkiewicz integral operator $\mathcal{M} _{h,\Omega , \mathcal{P},\varphi }^{\rho }$ on $\mathbb{R}^{d}$ by

1.10 $$ \mathcal{M}_{h,\Omega ,\mathcal{P},\varphi }^{\rho }f(x)= \biggl( \int _{0}^{\infty } \biggl\vert \frac{1}{t^{\rho }} \int_{ \vert y \vert \leq t}\frac{\Omega (y)h( \vert y \vert )}{ \vert y \vert ^{n- \rho }}f \bigl(x-\mathcal{P} \bigl(\varphi \bigl( \vert y \vert \bigr)y' \bigr) \bigr)\,dy \biggr\vert ^{2}\frac{dt}{t} \biggr)^{1/2}. $$

Our main result can be listed as follows.

### Theorem 1.1

*Let* $\mathcal{P}= (P_{1},P_{2}, \ldots ,P_{d})$ *be a polynomial mapping from* $\mathbb{R}^{n}$ *into* $\mathbb{R}^{d}$ *and* $\varphi \in \mathfrak{F}$, *where* $\mathfrak{F}$ *is the set of all functions* *ϕ* *satisfying the following conditions*:

- *ϕ* *is a positive increasing* $\mathcal{C}^{1} ( \mathbb{R}_{+})$ *function*;
- *there exist* $C_{\phi }$, $c_{\phi }>0$ *such that* $t\phi '(t) \geq C_{\phi }\phi (t)$ *and* $\phi (2t)\leq c_{\phi }\phi (t)$ *for all* $t>0$.

*Suppose that* $\Omega \in H^{1}({\rm S}^{n-1})$ *satisfies* (1.7) *and* $h\in \Delta_{\gamma }(\mathbb{R}_{+})$ *for some* $\gamma >1$. *Let* $\delta_{\gamma }=\max \{2, \gamma '\}$. *Then*

(i) *for* $\alpha \in (0,1)$ *and* $(1/p,1/q)\in \mathcal{R}_{ \gamma }\cup \{(1/p,1/p):\vert {1}/{p}-{1}/{2} \vert <1/\delta_{\gamma }\}$, *there exists a constant* $C>0$ *such that*

*where* $\mathcal{R}_{\gamma }$ *is the set of all interiors of the convex hull of three squares* $({1}/{2},{1}/{2}+ {1}/\delta_{\gamma })^{2}$, $({1}/{2}-{1}/\delta_{\gamma }, {1}/{2})^{2}$ *and* $({1}/{(2\gamma )},1- {1}/{(2\gamma )})^{2}$. *Moreover*, *the operator* $\mathcal{M}_{h,\Omega ,\mathcal{P}, \varphi }^{\rho }$ *is continuous from* $F_{\alpha }^{p,q} (\mathbb{R}^{d})$ *to* $\dot{F}_{\alpha }^{p,q}(\mathbb{R}^{d})$.
(ii) *for* $\alpha \in (0,1)$, $\vert {1}/{p}-{1}/{2} \vert <1/\delta_{ \gamma }$ *and* $q\in (1,\infty )$, *there exists a constant* $C>0$ *such that*

*Moreover*, *the operator* $\mathcal{M}_{h,\Omega ,\mathcal{P}, \varphi } ^{\rho }$ *is continuous from* $B_{\alpha }^{p,q} (\mathbb{R}^{d})$ *to* $\dot{B}_{\alpha }^{p,q}(\mathbb{R}^{d})$.

*The constants* *C* *may depend on* *α*, *ρ*, *p*, *q*, *n*, *d*, *φ* *and* $\deg (\mathcal{P})=\max_{1\leq i\leq d} \deg (P_{j})$, *but they are independent of the coefficients of* $\{P_{j}\}$.

### Remark 1.1

It was proved in [34] that the operator $\mathcal{M}_{h,\Omega ,\mathcal{P}, \varphi }^{\rho }$ is of type $(p,p)$ for $\vert {1}/{p}- {1}/{2} \vert <\min \{{1}/{2},{1}/{\gamma '} \}$ under the same conditions of Theorem 1.1. We observe that

1.11 $$ \bigl\vert \mathcal{M}_{h,\Omega ,\mathcal{P},\varphi }^{\rho }f-\mathcal{M} _{h,\Omega ,\mathcal{P},\varphi }^{\rho }g \bigr\vert \leq \bigl\vert \mathcal{M}_{h, \Omega ,\mathcal{P},\varphi }^{\rho }(f-g) \bigr\vert $$

for arbitrary functions *f*, *g* defined on $\mathbb{R}^{d}$. Combining (1.11) with the $L^{p}$ bounds for $\mathcal{M}_{h, \Omega , \mathcal{P},\varphi }^{\rho }$ shows that $\mathcal{M}_{h,\Omega , \mathcal{P},\varphi }^{\rho }$ is continuous on $L^{p}(\mathbb{R}^{d})$ for $\vert {1}/{p}-{1}/{2} \vert <\min \{{1}/{2},{1}/{\gamma '}\}$ under the same conditions of Theorem 1.1.

### Remark 1.2

We remark that the set $\mathcal{R} _{\gamma }$ was originally given by Yabuta [10] in the study of the boundedness for Marcinkiewicz integrals associated to surfaces $\{\varphi (\vert y \vert )y':y\in \mathbb{R}^{n}\}$ with $\varphi \in \mathfrak{F}$ on Triebel–Lizorkin spaces. Actually, Theorem 1.1 extends the partial result of [10, Theorem 1.1], which corresponds to the case $n=d$, $\rho >0$ and $\mathcal{P}(y)=y$. Clearly, $\mathcal{R} _{\gamma_{1}}\subsetneq \mathcal{R}_{\gamma_{2}}$ for any $1<\gamma _{1}<\gamma_{2}\leq \infty $ and $\mathcal{R}_{\infty }=(0,1)\times (0,1)$. There are some model examples for the class $\mathfrak{F}$, such as $t^{\alpha }$ ($\alpha >0$), $t^{\beta }\ln (1+t)$ ($\beta \geq 1$), $t\ln \ln (e+t)$, real-valued polynomials *P* on $\mathbb{R}$ with positive coefficients and $P(0)=0$ and so on. Note that there exists $B_{\varphi }>1$ such that $\varphi (2t) \geq B_{\varphi }\varphi (t)$ for any $\varphi \in \mathfrak{F}$ (see [7]).

By the Properties (1.5) and (1.6), Remark 1.1 and Theorem 1.1, we can get the following result immediately.

### Theorem 1.2

*Under the same conditions of Theorem* 1.1, *the operator* $\mathcal{M}_{h,\Omega ,\mathcal{P}, \varphi }^{\rho }$ *is bounded and continuous on* $F_{\alpha }^{p,q}( \mathbb{R}^{d})$ *and* $B_{\alpha }^{p,q}(\mathbb{R}^{d})$, *respectively*.

### Remark 1.3

Since $L^{\infty }(\mathbb{R}_{+}) \subsetneq \Delta_{\gamma }(\mathbb{R}_{+})$ for any $1<\gamma < \infty $ and $L^{r}(\mathrm{S}^{n-1}) \subsetneq H^{1}(\mathrm{S}^{n-1})$ for any $r>1$, the boundedness part in Theorem 1.2 improves and generalizes greatly the results of [12, 13]. It should be pointed out that our main results are new even in the special case: $\rho =1$, $n=d$, $h(t)\equiv 1$ and $\varphi (t)=t$.

The paper is organized as follows. Section 2 contains two vector-valued inequalities on maximal functions, which are the main ingredients of our proofs. Section 3 is devoted to presenting some preliminary lemmas. The proof of Theorem 1.2 will be given in Sect. 4. We would like to remark that some ideas in our proofs are taken from [7, 10, 17, 23, 34] and the main novelty in this paper is to give the continuity for Marcinkiewicz integral operators on Triebel–Lizorkin spaces and Besov spaces.

Throughout this note, we denote by $p'$ the conjugate index of *p*, i.e. ${1}/{p}+{1}/{p'}=1$. The letter *C* or *c*, sometimes with certain parameters, will stand for positive constants not necessarily the same one at each occurrence, but are independent of the essential variables. If $f\leq Cg$, we then write $f\lesssim g$ or $g\gtrsim f$; and if $f\lesssim g\lesssim f$, we then write $f\thicksim g$. In what follows, we denote by $J^{-1}$ and $J^{t}$ the inverse transform and the transpose of the linear transformation *J*, respectively. We also denote the Dirac delta function on $\mathbb{R}^{d}$ by $\delta_{\mathbb{R} ^{d}}$. For $m\leq d$, we denote the projection operator from $\mathbb{R}^{m}$ to $\mathbb{R}^{d}$ by $\pi_{m}^{d}$. We set $\mathfrak{R}_{d}=\{\xi \in \mathbb{R}^{d}; 1/2<\vert \xi \vert \leq 1\}$. We also use the conventions $\sum_{i\in \emptyset }a_{i}=0$ and $\prod_{i\in \emptyset }a_{i}=1$.

*Comments on conclusions and methods.* This aim of this paper is to investigate the boundedness and continuity for the parametric Marcinkiewicz integral operators supported by polynomial compound mappings $\mathcal{M}_{h, \Omega ,\mathcal{P},\varphi }^{ \rho }$ on the Triebel–Lizorkin spaces and Besov spaces. This is motivated by some recent results (see [4, 10, 11, 25, 31]). In [4], the authors established the bounds for the singular integral operators supported by polynomial mappings on the Triebel–Lizorkin spaces and Besov spaces; In [10, 11] the authors proved the boundedness for Marcinkiewicz integral operators $\mathcal{M}_{h,\Omega }^{\rho }$ on the Triebel–Lizorkin spaces; In [25, 31] the authors gave the $L^{p}$ bounds for the Marcinkiewicz integral operators supported by polynomial mappings $\mathcal{M}_{h,\Omega ,\mathcal{P}}$. The main purpose of this paper will not only address the residual problems with respect to exponents [25, 31] but also establish the corresponding continuity of Marcinkiewicz integral operators on Triebel–Lizorkin spaces and Besov spaces. Although the methods and idea used in proofs of main results are motivated by some previous work [7, 10, 16, 22, 31], the methods and techniques are more delicate and difficult than those in the above references. Moreover, the main results are new and the proofs are highly non-trivial. On the other hand, the main results greatly extended and generalized some previous work [10–12].

## Two vector-valued inequalities on maximal functions

The following lemma can be seen as a general case of [10, Lemma 6.1], which can be proved by [20, Theorem 4.6.1] and [20, Proposition 4.6.4]. We omit the details.

### Lemma 2.1

*Let* $\mathcal{B}_{1}$, $\mathcal{B}_{2}$ *be two Banach spaces and* $\rho (\cdot )$ *denote the corresponding norm of* $\mathbb{R}^{d}$. *Let* *T⃗* *be a bounded linear operator from* $L^{p_{0}} (\mathcal{B}_{1},\mathbb{R}^{d})$ *to* $L^{p_{0}}(\mathcal{B}_{2}, \mathbb{R}^{d})$ *with norm* $A>0$ *for some* $1< p_{0}\leq \infty $, *for which there exists a kernel* *K⃗* *defined on* $\mathbb{R}^{d}\backslash \{0\}$ *that takes values in the space* $\mathcal{L}(\mathcal{B}_{1},\mathcal{B}_{2})$ *such that*

$$ \vec{T}(F) (x)= \int_{\mathbb{R}^{d}}\vec{K}(x-y)F(y)\,dy, $$

*is well*-*defined as an element of* $\mathcal{B}_{2}$ *for all* $L^{\infty }(\mathcal{B}_{1},\mathbb{R}^{d})$ *functions* *F* *with compact supported provided* *x* *lies outside the support of F*. *Assume that the kernel* *K⃗* *satisfies Hörmander condition*

$$\underset{y\in R^{d}\setminus \{0\}}{\sup}\int_{\rho (x)\geq 2\rho (y)}{∥\vec{K}(x-y)-\vec{K}(x)∥}_{B_{1}\to B_{2}}\;dx=B<\infty .$$

*Then*, *for any* $1< p$, $q<\infty $ *and all* $\mathcal{B}_{1}$-*valued functions* $F_{j}$, *there exists* $C>0$, *such that*

$${∥{\left(\sum_{j\in Z}{∥\vec{T}(F_{j})∥}_{B_{2}}^{q}\right)}^{1/q}∥}_{L^{p}(R^{d})}≲(A+B){∥{\left(\sum_{j\in Z}{∥F_{j}∥}_{B_{1}}^{q}\right)}^{1/q}∥}_{L^{p}(R^{d})}.$$

We now establish the following vector-valued inequality of a Hardy–Littlewood maximal function, which is of interest in its own right.

### Lemma 2.2

*Let* $\mathrm{M}_{(d)}$ *be the Hardy–Littlewood maximal operator defined on* $\mathbb{R}^{d}$. *Then*

$$\begin{array}{c} {∥{\left(\sum_{k\in Z}{∥{\left(\sum_{j\in Z}|M_{\left(d\right)}(g_{j,\zeta ,k}){|}^{s}\right)}^{1/s}∥}_{L^{r}(R_{d})}^{q}\right)}^{1/q}∥}_{L^{p}(R^{d})}\\ \;≲{∥{\left(\sum_{k\in Z}{∥{\left(\sum_{j\in Z}|g_{j,\zeta ,k}{|}^{s}\right)}^{1/s}∥}_{L^{r}(R_{d})}^{q}\right)}^{1/q}∥}_{L^{p}(R^{d})} \end{array}$$

*for all* $1< p,q,r,s<\infty $.

### Proof

Let Φ be a positive radial symmetrically decreasing Schwartz function on $\mathbb{R}^{d}$ such that $\Phi (x) \geq 1$ when $\vert x \vert \leq 1$. Let $\Phi_{t}(x)=t^{-d}\Phi (\frac{x}{t})$ for all $t>0$ and $\mathrm{M}_{\Phi }^{d}(f)=\sup_{k\in \mathbb{Z}}\vert f*\Phi_{2^{k}} \vert $. As in [20, p. 336] we have

2.1 $$ \mathrm{M}_{(d)}(f) (x)\leq 2^{d}\mathrm{M}_{\Phi }^{d} \bigl( \vert f \vert \bigr) (x)\lesssim \mathrm{M}_{(d)}(f) (x) \quad \forall x\in \mathbb{R}^{d}. $$

Let $\mathcal{B}_{1}=L^{r}(\ell^{s},\mathfrak{R}_{d})$ and $\mathcal{B}_{2}=L^{r}(\ell^{\infty }(\ell^{s}),\mathfrak{R}_{d})$ with $1< r$, $s<\infty $. Define the operator $\overrightarrow{{\mathrm{M} _{\Phi }^{d}}}$ by

$$ \overrightarrow{{\mathrm{M}_{\Phi }^{d}}}(F) (x)=\vec{K}*F(x)= \bigl\{ \Phi_{2^{l}}*F(x) \bigr\} _{l\in \mathbb{Z}} \quad \mbox{with } F\in L^{r} \bigl( \mathcal{B}_{1},\mathbb{R}^{d} \bigr). $$

(2.1) together with the $L^{r}(\ell^{s},\mathbb{R}^{d})$-boundedness of the Hardy–Littlewood maximal functions and Fubini’s theorem shows that

$$\begin{array}{rl} {∥{∥\vec{M_{\Phi }^{d}}\left(\{f_{j,\zeta }\}\right)(x)∥}_{B_{2}}∥}_{L^{r}(R^{d})}^{r} & =\int_{R^{d}}\int_{R_{d}}{\left(\underset{l\in Z}{\sup}{\left(\sum_{j\in Z}|\Phi_{2^{l}}*f_{j,\zeta }(x){|}^{s}\right)}^{1/s}\right)}^{r}\;d\zeta \;dx\\ & ≲\int_{R_{d}}\int_{R^{d}}{\left(\sum_{j\in Z}|M_{\left(d\right)}(f_{j,\zeta })(x){|}^{s}\right)}^{r/s}\;dx\;d\zeta \\ & ≲\int_{R_{d}}\int_{R^{d}}{\left(\sum_{j\in Z}|f_{j,\zeta }(x){|}^{s}\right)}^{r/s}\;dx\;d\zeta \\ & ≲{∥{∥\{f_{j,\zeta }\}∥}_{B_{1}}∥}_{L^{r}(R^{d})}^{r}, \end{array}$$

which implies that $\overrightarrow{\mathrm{M}_{\Phi }^{d}}$ is bounded from $L^{r}(\mathcal{B}_{1},\mathbb{R}^{d})$ to $L^{r}(\mathcal{B} _{2},\mathbb{R}^{d})$. On the other hand, for any $x,y\in \mathbb{R}^{d}$,

2.2 $$\begin{array}{rl} & {∥\left(\vec{K}(x-y)-\vec{K}(x)\right)\left(\left\{f_{j,\zeta }(x)\right\}\right)∥}_{B_{2}}\\ & \;={\left(\int_{R_{d}}{\left(\underset{l\in Z}{\sup}{\left(\sum_{j\in Z}|\left(\Phi_{2^{l}}(x-y)-\Phi_{2^{l}}(x)\right)f_{j,\zeta }(x){|}^{s}\right)}^{1/s}\right)}^{r}\;d\zeta \right)}^{1/r}\\ & \;={\left(\int_{R_{d}}{\left(\underset{k\in Z}{\sup}|\Phi_{2^{l}}(x-y)-\Phi_{2^{l}}(x)|{\left(\sum_{j\in Z}|f_{j,\zeta }{|}^{s}\right)}^{1/s}\right)}^{r}\;d\zeta \right)}^{1/r}\\ & \;\leq \underset{l\in Z}{\sup}|\Phi_{2^{l}}(x-y)-\Phi_{2^{l}}(x)|{∥\left\{f_{j,\zeta }(x)\right\}∥}_{B_{1}}. \end{array}$$

From [20, (4.6.19)] we have

$$ \sup_{y\in \mathbb{R}^{d}\backslash \{0\}} \int_{ \vert x \vert \geq 2 \vert y \vert } \sup_{l\in \mathbb{Z}} \bigl\vert \Phi_{2^{l}}(x-y)-\Phi_{2^{l}}(x) \bigr\vert \,dx \leq C_{d}< \infty . $$

This together with (2.2) yields

$$\underset{y\in R^{d}\setminus \{0\}}{\sup}\int_{\left|x|\geq 2|y\right|}{∥\vec{K}(x-y)-\vec{K}(x)∥}_{B_{1}\to B_{2}}\;dx\leq C_{d}<\infty .$$

Applying Lemma 2.1 with $\rho (\cdot )=\vert \cdot \vert $, we obtain

$${∥{\left(\sum_{k\in Z}{∥\vec{M_{\Phi }^{d}}\left(\{g_{j,\zeta ,k}\}\right)∥}_{B_{2}}^{q}\right)}^{1/q}∥}_{L^{p}(R^{d})}≲{∥{\left(\sum_{k\in Z}{∥\{g_{j,\zeta ,k}\}∥}_{B_{1}}^{q}\right)}^{1/q}∥}_{L^{p}(R^{d})}$$

for any $1< p$, $q<\infty $. This proves Lemma 2.2. □

We end this section by presenting the following lemma, which plays a key role in the proof of Theorem 1.1.

### Lemma 2.3

([17])

*Let* $\mathcal{P}=(P _{1},P_{2},\ldots ,P_{d})$ *be a polynomial mapping from* $\mathbb{R} ^{n}$ *into* $\mathbb{R}^{d}$ *and* $\mathrm{M}_{\mathcal{P}}$ *denote the Hardy–Littlewood maximal operator associated to* $\mathcal{P}$ *defined by*

$$ \mathrm{M}_{\mathcal{P}}(f) (x)=\sup_{r>0}\frac{1}{r^{n}} \int_{ \vert y \vert \leq r} \bigl\vert f \bigl(x-\mathcal{P}(y) \bigr) \bigr\vert \,dy. $$

*Then*, *for any* $1< p,q,r<\infty $, *there exists a constant* $C>0$ *independent of the coefficients of* $\{P_{j}\}$ *such that*

$${∥{\left(\sum_{j\in Z}{∥M_{P}(f_{j,\zeta })∥}_{L^{r}(R_{d})}^{q}\right)}^{1/q}∥}_{L^{p}(R^{d})}\leq C{∥{\left(\sum_{j\in Z}{∥f_{j,\zeta }∥}_{L^{r}(R_{d})}^{q}\right)}^{1/q}∥}_{L^{p}(R^{d})}.$$

## Preliminary notations and lemmas

Let $\mathcal{S}(\mathrm{S}^{n-1})$ be the Schwartz space of smooth functions on $\mathrm{S}^{n-1}$ and $\mathcal{S}'(\mathrm{S}^{n-1})$ denote its dual. For $f\in \mathcal{S}'$, we define the radial maximal function $P^{+}f$ by

$$ P^{+}f(w)=\sup_{0\leq r< 1} \biggl\vert \int_{\mathrm{S}^{n-1}}\Omega ( \theta )\frac{1-r^{2}}{ \vert rw-\theta \vert ^{n}}\,d\sigma (\theta ) \biggr\vert . $$

The Hardy space $H^{1}(\mathrm{S}^{n-1})$ is defined by

$$H^{1}\left(S^{n-1}\right)=\left\{f\in S^{\prime }\left(S^{n-1}\right):{∥f∥}_{H^{1}(S^{n-1})}={∥P^{+}f∥}_{L^{1}(S^{n-1})}<\infty \right\}.$$

Let us recall the definition of atoms.

### Definition 3.1

A function $a(\cdot )$ on $\mathrm{S}^{n-1}$ is a regular atom if there exist $\varepsilon \in {\mathrm{S}}^{n-1}$ and $\varrho \in (0,2]$ such that

3.1 $$\begin{aligned}& \operatorname{supp} (a)\subset {\mathrm{S}}^{n-1}\cap B(\varepsilon , \varrho ), \quad \mbox{where } B(\varepsilon ,\varrho )= \bigl\{ y\in \mathbb{R}^{n}: \vert y- \varepsilon \vert < \varrho \bigr\} ; \end{aligned}$$

3.2 $${∥a∥}_{L^{\infty }(S^{n-1})}\leq \varrho^{-n+1};$$

3.3 $$\begin{aligned}& \int_{\mathrm{S}^{n-1}}a(y)\,d\sigma (y)=0. \end{aligned}$$

The following lemma is the well-known atomic decomposition of Hardy space (see [35, 36]).

### Lemma 3.1

*For any* $\Omega \in H^{1}(\mathrm{S} ^{n-1})$ *satisfying* (1.1), *there are complex numbers* $\{c_{j}\}$ *and regular atoms* $\{\Omega_{j}\}$ *such that* $\Omega =\sum_{j}c_{j}\Omega _{j}$ *and* ${∥\Omega ∥}_{H^{1}(S^{n-1})}∼\sum_{j}|c_{j}|$.

Let *h*, Ω, *ρ* be given as in (1.3). For $t>0$ and a mapping $\Gamma :\mathbb{R}^{n} \rightarrow \mathbb{R}^{d}$, we define the measures $\{\sigma_{h,\Omega ,\Gamma ,t,\rho }\}_{t>0}$ on $\mathbb{R}^{d}$ by

$$ \int_{\mathbb{R}^{d}}f\,d\sigma_{h,\Omega ,\Gamma ,t,\rho }=\frac{1}{t^{\rho }} \int_{t/2< \vert y \vert \leq t}f \bigl(\Gamma (y) \bigr)\frac{\Omega (y)h( \vert y \vert )}{ \vert y \vert ^{n- \rho }}\,dy. $$

We also define $\sigma_{h,\Omega ,\Gamma ,\rho }^{*}$ on $\mathbb{R} ^{d}$ by

$$ \sigma_{h,\Omega ,\Gamma ,\rho }^{*}(f) (y)=\sup_{t>0} \bigl\vert \vert \sigma_{h,\Omega ,\Gamma ,t,\rho } \vert *f(y) \bigr\vert , $$

where $\vert \sigma_{h,\Omega ,\Gamma ,t,\rho } \vert $ is defined in the same way as $\sigma_{h,\Omega ,\Gamma ,t,\rho }$, but with *h* and Ω replaced by $\vert h \vert $ and $\vert \Omega \vert $, respectively.

### Lemma 3.2

*Let* $\Gamma (y)= \mathcal{P}( \varphi (\vert y \vert )y')$ *with* $\varphi \in \mathfrak{F}$ *and* $\mathcal{P}=(P _{1},P_{2},\ldots ,P_{d})$ *being a polynomial mapping from* $\mathbb{R}^{n}$ *into* $\mathbb{R}^{d}$. *Suppose that* $h\in \Delta_{ \gamma }(\mathbb{R}_{+})$ *for some* $\gamma >1$ *and* $\Omega \in L^{1}( \mathrm{S}^{n-1})$. *Then*, *for* $(1/p,1/q,1/r)\in \mathcal{Q}_{\gamma }$, *there exists a constant* $C>0$ *independent of the coefficients of* $\{P_{j}\}$ *such that*

3.4 $$\begin{array}{rl} & {∥{\left(\sum_{j\in Z}{\left(\int_{R_{d}}{\left(\sum_{k\in Z}\int_{1}^{2}||\sigma_{h,\Omega ,\Gamma ,t,\rho }|*g_{j,\zeta ,k}{|}^{2}\frac{dt}{t}\right)}^{1/2}\;d\zeta \right)}^{q}\right)}^{1/q}∥}_{L^{p}(R^{d})}\\ & \;\leq C{∥h∥}_{\Delta_{\gamma }(R_{+})}{∥\Omega ∥}_{L^{1}(S^{n-1})}{∥{\left(\sum_{j\in Z}{∥{\left(\sum_{k\in Z}|g_{j,\zeta ,k}{|}^{2}\right)}^{1/2}∥}_{L^{r}(R_{d})}^{q}\right)}^{1/q}∥}_{L^{p}(R^{d})} \end{array}$$

*holds for functions* $\{g_{j,\zeta ,k}\}_{j,\zeta ,k}\in L^{p} (\ell ^{q}(L^{r}(\ell^{2})),\mathbb{R}^{d})$, *where* $\mathcal{Q}_{\gamma }$ *is the set of all interiors of the convex hull of three cubes* $(\frac{1}{2},\frac{1}{2}+ \frac{1}{\max \{2,\gamma '\}})^{3}$, $(\frac{1}{2}-\frac{1}{\max \{2,\gamma '\}},\frac{1}{2})^{3}$, *and* $(\frac{1}{2\gamma },1-\frac{1}{2\gamma })^{3}$.

### Proof

To prove (3.4), it suffices to show that there exists a constant $C>0$ independent of the coefficients of $\{P_{j}\}$ such that

3.5 $$\begin{array}{rl} & {∥{\left(\sum_{j\in Z}{∥{\left(\sum_{k\in Z}\int_{1}^{2}||\sigma_{h,\Omega ,\Gamma ,t,\rho }|*g_{j,\zeta ,k}{|}^{2}\frac{dt}{t}\right)}^{1/2}∥}_{L^{r}(R_{d})}^{q}\right)}^{1/q}∥}_{L^{p}(R^{d})}\\ & \;\leq C{∥h∥}_{\Delta_{\gamma }(R_{+})}{∥\Omega ∥}_{L^{1}(S^{n-1})}{∥{\left(\sum_{j\in Z}{∥{\left(\sum_{k\in Z}|g_{j,\zeta ,k}{|}^{2}\right)}^{1/2}∥}_{L^{r}(R_{d})}^{q}\right)}^{1/q}∥}_{L^{p}(R^{d})} \end{array}$$

holds for functions $\{g_{j,\zeta ,k}\}_{j,\zeta ,k}\in L^{p}(\ell ^{q}(L^{r}(\ell^{2},\mathfrak{R}_{d})),\mathbb{R}^{d})$ with $(1/p,1/q,1/r)\in \mathcal{Q}_{\gamma }$. By the change of variables and Hölder’s inequality,

$$\begin{array}{rl} & \sigma_{h,\Omega ,\Gamma ,\rho }^{*}(f)(x)\\ & \;\leq \underset{t>0}{\sup}\int_{t/2<|y|\leq t}|f\left(x-\Gamma (y)\right)|\frac{\left|h(|y|)\Omega (y)\right|}{|y{|}^{n}}\;dy\\ & \;=\underset{t>0}{\sup}\int_{t/2}^{t}\int_{S^{n-1}}|f\left(x-\Gamma (r\theta )\right)||\Omega (\theta )|\;d\sigma (\theta )|h(r)|\frac{dr}{r}\\ & \;\leq 2{∥h∥}_{\Delta_{\gamma }(R_{+})}{∥\Omega ∥}_{L^{1}(S^{n-1})}^{1/\gamma }{\left(\int_{S^{n-1}}\underset{t>0}{\sup}\int_{t/2}^{t}|f\left(x-\Gamma (r\theta )\right){|}^{\gamma^{\prime }}\frac{dr}{r}|\Omega (\theta )|\;d\sigma (\theta )\right)}^{1/\gamma^{\prime }}\\ & \;\leq C{∥h∥}_{\Delta_{\gamma }(R_{+})}{∥\Omega ∥}_{L^{1}(S^{n-1})}^{1/\gamma }\\ & \;\;\times {\left(\int_{S^{n-1}}\underset{t>0}{\sup}\int_{\varphi (t/2)}^{\varphi (t)}|f\left(x-\Gamma \left(\varphi^{-1}(s)\theta \right)\right){|}^{\gamma^{\prime }}\frac{ds}{\varphi^{-1}(s)\varphi^{\prime }(\varphi^{-1}(s))}|\Omega (\theta )|\;d\sigma (\theta )\right)}^{1/\gamma^{\prime }}\\ & \;\leq C(\varphi ){∥h∥}_{\Delta_{\gamma }(R_{+})}{∥\Omega ∥}_{L^{1}(S^{n-1})}^{1/\gamma }\\ & \;\;\times {\left(\int_{S^{n-1}}\underset{t>0}{\sup}\frac{1}{t}\int_{|s|\leq t}|f\left(x-\Gamma \left(\varphi^{-1}(s)\theta \right)\right){|}^{\gamma^{\prime }}\;ds|\Omega (\theta )|\;d\sigma (\theta )\right)}^{1/\gamma^{\prime }}, \end{array}$$

which together with Lemma 2.3 and Minkowski’s inequality shows that

3.6 $$\begin{array}{rl} & {∥{\left(\sum_{j\in Z}{∥\sigma_{h,\Omega ,\Gamma ,\rho }^{*}(f_{j,\zeta })∥}_{L^{r}(R_{d})}^{q}\right)}^{1/q}∥}_{L^{p}(R^{d})}\\ & \;\leq C{∥h∥}_{\Delta_{\gamma }(R_{+})}{∥\Omega ∥}_{L^{1}(S^{n-1})}{∥{\left(\sum_{j\in Z}{∥f_{j,\zeta }∥}_{L^{r}(R_{d})}^{q}\right)}^{1/q}∥}_{L^{p}(R^{d})} \end{array}$$

for any $\gamma '< p,q,r<\infty $. Here $C>0$ is independent of *h*, Ω and the coefficients of $\{P_{j}\}$.

We now prove (3.5) by considering the following three cases:

*Case 1* $(1<\gamma \leq \infty )$. By the duality argument, Hölder’s inequality, Fubini’s theorem and (3.6), we have, for any $1< p,q,r<\gamma $, there exist functions $\{f_{j,\zeta }\}_{j, \zeta }$ with ${∥\{f_{j,\zeta }\}∥}_{L^{p^{\prime }}(\ell^{q^{\prime }}(L^{r^{\prime }}(R_{d})),R^{d})}=1$ such that

3.7 $$\begin{array}{rl} & {∥{\left(\sum_{j\in Z}{∥\sum_{k\in Z}\int_{1}^{2}||\sigma_{h,\Omega ,\Gamma ,t,\rho }|*g_{j,\zeta ,k}|\frac{dt}{t}∥}_{L^{r}(R_{d})}^{q}\right)}^{1/q}∥}_{L^{p}(R^{d})}\\ & \;=\sum_{j\in Z}\int_{R^{d}}\int_{R_{d}}\sum_{k\in Z}\int_{1}^{2}||\sigma_{h,\Omega ,\Gamma ,t,\rho }|*g_{j,\zeta ,k}(x)|\frac{dt}{t}|f_{j,\zeta }(x)|\;d\zeta dx\\ & \;\leq \sum_{j\in Z}\int_{R^{d}}\int_{R_{d}}\sum_{k\in Z}|g_{j,\zeta ,k}(x)|\int_{1}^{2}|\sigma_{h,\Omega ,\Gamma ,t,\rho }|*\tilde{\left|f_{j,\zeta }\right|}(-x)\frac{dt}{t}\;d\zeta \;dx\\ & \;\leq \sum_{j\in Z}\int_{R^{d}}\int_{R_{d}}\sum_{k\in Z}|g_{j,\zeta ,k}(x)|\sigma_{h,\Omega ,\Gamma ,\rho }^{*}\left(\tilde{\left|f_{j,\zeta }\right|}\right)(-x)\;d\zeta dx\\ & \;\leq {∥{\left(\sum_{j\in Z}{∥\sum_{k\in Z}|g_{j,\zeta ,k}|∥}_{L^{r}(R_{d})}^{q}\right)}^{1/q}∥}_{L^{p}(R^{d})}{∥{\left(\sum_{j\in Z}{∥\sigma_{h,\Omega ,\Gamma ,\rho }^{*}\left(\tilde{\left|f_{j,\zeta }\right|}\right)∥}_{L^{r^{\prime }}(R_{d})}^{q^{\prime }}\right)}^{1/q^{\prime }}∥}_{L^{p^{\prime }}(R^{d})}\\ & \;≲{∥h∥}_{\Delta_{\gamma }(R_{+})}{∥\Omega ∥}_{L^{1}(S^{n-1})}{∥{\left(\sum_{j\in Z}{∥\sum_{k\in Z}|g_{j,\zeta ,k}|∥}_{L^{r}(R_{d})}^{q}\right)}^{1/q}∥}_{L^{p}(R^{d})}, \end{array}$$

where $\widetilde{f_{j,\zeta }}(x)=f_{j,\zeta }(-x)$. On the other hand, it follows from (3.6) that

3.8 $$\begin{array}{rl} & {∥{\left(\sum_{j\in Z}{∥\underset{k\in Z}{\sup}\underset{t\in [1,2]}{\sup}||\sigma_{h,\Omega ,\Gamma ,t,\rho }|*g_{j,\zeta ,k}|∥}_{L^{r}(R_{d})}^{q}\right)}^{1/q}∥}_{L^{p}(R^{d})}\\ & \;\leq {∥{\left(\sum_{j\in Z}{∥\sigma_{h,\Omega ,\Gamma ,\rho }^{*}\left(\underset{k\in Z}{\sup}|g_{j,\zeta ,k}|\right)∥}_{L^{r}(R_{d})}^{q}\right)}^{1/q}∥}_{L^{p}(R^{d})}\\ & \;\leq C{∥h∥}_{\Delta_{\gamma }(R_{+})}{∥\Omega ∥}_{L^{1}(S^{n-1})}{∥{\left(\sum_{j\in Z}{∥\underset{k\in Z}{\sup}|g_{j,\zeta ,k}|∥}_{L^{r}(R_{d})}^{q}\right)}^{1/q}∥}_{L^{p}(R^{d})} \end{array}$$

for any $\gamma '< p,q,r<\infty $. Interpolating between (3.7) and (3.8) shows that (3.5) holds for $(1/p,1/q,1/r)$ belonging to the interior of the cube $(\frac{1}{2\gamma }, 1-\frac{1}{2\gamma })^{3}$.

*Case 2* $(1<\gamma \leq 2)$. By Hölder’s inequality, we have

$$\begin{array}{rl} & \left||\sigma_{h,\Omega ,\Gamma ,t,\rho }|*g_{j,\zeta ,k}(x)\right|\\ & \;\leq \int_{t/2<|y|\leq t}|g_{j,\zeta ,k}\left(x-\Gamma (y)\right)|\frac{\left|h(y)\Omega (y)\right|}{|y{|}^{n}}\;dy\\ & \;\leq {\left(\int_{t/2<|y|\leq t}|g_{j,\zeta ,k}\left(x-\Gamma (y)\right){|}^{2}\frac{\left|h(y){|}^{2-\gamma }|\Omega (y)\right|}{|y{|}^{n}}\;dy\right)}^{1/2}{\left(\int_{t/2<|y|\leq t}\frac{\left|h(y){|}^{\gamma }|\Omega (y)\right|}{|y{|}^{n}}\;dy\right)}^{1/2}\\ & \;\leq C{∥h∥}_{\Delta_{\gamma }(R_{+})}{∥\Omega ∥}_{L^{1}(S^{n-1})}^{1/2}{\left(|\sigma_{|h{|}^{2-\gamma },\Omega ,\Gamma ,t,\rho }|*|g_{j,\zeta ,k}{|}^{2}(x)\right)}^{1/2}. \end{array}$$

It follows that

3.9 $$\begin{array}{rl} & {∥{\left(\sum_{j\in Z}{∥{\left(\sum_{k\in Z}\int_{2^{kv}}^{2^{(k+1)v}}||\sigma_{h,\Omega ,\Gamma ,t,\rho }|*g_{j,\zeta ,k}{|}^{2}\frac{dt}{t}\right)}^{1/2}∥}_{L^{r}(R_{d})}^{q}\right)}^{1/q}∥}_{L^{p}(R^{d})}\\ & \;\leq {∥h∥}_{\Delta_{\gamma }(R_{+})}{∥\Omega ∥}_{L^{1}(S^{n-1})}^{1/2}\\ & \;\;\times {∥{\left(\sum_{j\in Z}{∥\sum_{k\in Z}\int_{1}^{2}|\sigma_{|h{|}^{2-\gamma },\Omega ,\Gamma ,t}|*|g_{j,\zeta ,k}{|}^{2}\frac{dt}{t}∥}_{L^{r}(R_{d})}^{q}\right)}^{1/q}∥}_{L^{p}(R^{d})}. \end{array}$$

Observe that ${∥|h{|}^{2-\gamma }∥}_{\Delta_{\gamma /(2-\gamma )}(R_{+})}\leq C{∥h∥}_{\Delta_{\gamma }(R_{+})}$. By (3.9) and (3.7) with *γ*, *p*, *q*, *r* replacing by $\frac{\gamma }{2-\gamma }$, $\frac{p}{2}$, $\frac{q}{2}$, $\frac{r}{2}$, respectively we have (3.5) for $(1/p,1/q,1/r)$ belonging to the interior of the cube $(\frac{1}{2}- \frac{1}{\gamma '},\frac{1}{2})^{3}$. By duality, (3.5) also holds for $(1/p,1/q,1/r)$ belonging to the interior of the cube $(\frac{1}{2},\frac{1}{2}+\frac{1}{\gamma '})^{3}$. Interpolating these two cases, we see that (3.5) holds for $(1/p,1/q,1/r)$ belonging to the interior of the convex hull of two cubes $(\frac{1}{2}-\frac{1}{ \gamma '},\frac{1}{2})^{3}$ and $(\frac{1}{2},\frac{1}{2}+\frac{1}{ \gamma '})^{3}$. We notice that the interior of the cubes $(\frac{1}{2 \gamma }, 1-\frac{1}{2\gamma })^{3}$ contains in the interior of the convex hull of two cubes $(\frac{1}{2}-\frac{1}{\gamma '}, \frac{1}{2})^{3}$ and $(\frac{1}{2},\frac{1}{2}+ \frac{1}{\gamma '})^{3}$ when $1<\gamma \leq 2$.

*Case 3* $(\gamma \geq 2)$. Clearly, ${∥h∥}_{\Delta_{2}(R_{+})}\leq {∥h∥}_{\Delta_{\gamma }(R_{+})}$ for $\gamma \geq 2$. Interpolating between cases 1 and 2 we obtain (3.5) for $(1/p,1/q,1/r)$ belonging to the interior of the convex hull of three cubes $(\frac{1}{2\gamma },1-\frac{1}{2\gamma })^{3}$, $(0, \frac{1}{2})^{3}$ and $(\frac{1}{2},1)^{3}$. This completes the proof of Lemma 3.2. □

Let $\{b_{k}\}$ be a lacunary sequence such that $1<\delta_{1}\leq \frac{b _{k+1}}{b_{k}}\leq \delta_{2}$ for all $k\in \mathbb{Z}$. Let $\{\lambda_{k}\}_{k\in \mathbb{Z}}$ be a collection of $\mathcal{C} _{0}^{\infty }(\mathbb{R}_{+})$ with the following properties: $\operatorname{supp}(\lambda_{k}) \subset [b_{k}^{-1},b_{k-2}^{-1}]$, $0\leq \lambda_{k}(t)\leq 1$ and $\sum_{k\in \mathbb{Z}}\lambda_{k}(t)=1$. We have the following result.

### Lemma 3.3

*For* $m\leq d$, *let* $H:\mathbb{R} ^{m}\rightarrow \mathbb{R}^{m}$ *and* $H:\mathbb{R}^{d} \rightarrow \mathbb{R}^{d}$ *be two nonsingular linear transformations*. *Define the multiplier operator* $S_{k}$ *on* $\mathbb{R}^{d}$ *by*

$$ \widehat{S_{k}f}(\xi )=\lambda_{k} \bigl( \bigl\vert H \pi_{m}^{d}G\xi \bigr\vert \bigr)\hat{f}(\xi ). $$

*Then*, *for* $1< p,q,r<\infty $, *there exists a constant* $C>0$ *depending only on* $\delta_{2}$ *and* *d* *such that*

$${∥{\left(\sum_{j\in Z}{∥{\left(\sum_{k\in Z}|S_{k}f_{\zeta ,j}{|}^{2}\right)}^{1/2}∥}_{L^{r}(R_{d})}^{q}\right)}^{1/q}∥}_{L^{p}(R^{d})}\leq C{∥{\left(\sum_{j\in Z}{∥f_{\zeta ,j}∥}_{L^{r}(R_{d})}^{q}\right)}^{1/q}∥}_{L^{p}(R^{d})}.$$

### Proof

Define the operator $\vec{T}f: =\{\Phi_{k}*f \}_{k\in \mathbb{Z}}$ with $\widehat{\Phi _{k}} (\xi )=\lambda_{k}(\vert \xi \vert )$. We first prove that

3.10 $${∥{\left(\sum_{j\in Z}{∥{\left(\sum_{k\in Z}|\Phi_{k}*f_{j,\zeta }{|}^{2}\right)}^{1/2}∥}_{L^{r}(R_{d})}^{q}\right)}^{1/q}∥}_{L^{p}(R^{d})}≲{∥{\left(\sum_{j\in Z}{∥f_{j,\zeta }∥}_{L^{r}(R_{d})}^{q}\right)}^{1/q}∥}_{L^{p}(R^{d})}$$

for any $1< p,q,r<\infty $. One can easily check that $\sum_{k\in \mathbb{Z}}\vert \widehat{\Phi _{k}}(\xi ) \vert ^{2}\leq 1$ for all $\xi \neq 0$. By Plancherel’s theorem we see that *T⃗* is bounded from $L^{2}(\mathbb{R}^{d})$ to $L^{2}(\ell^{2},\mathbb{R}^{d})$. Next we shall prove that

3.11 $$ \int_{ \vert x \vert \geq 2 \vert y \vert } \biggl(\sum_{k\in \mathbb{Z}} \bigl\vert \Phi_{k}(x-y)- \Phi_{k}(x) \bigr\vert ^{2} \biggr)^{1/2}\,dx\leq C. $$

It is clear that

$$ (-2\pi ix)^{\alpha }\Phi_{k}(x)= \int_{\mathbb{R}^{d}}\partial^{\alpha }\lambda_{k} \bigl( \vert \xi \vert \bigr)e^{2\pi ix\cdot \xi }\,d\xi \quad \mbox{for any multi-index } \alpha . $$

Taking $\vert \alpha \vert =d+1$, we obtain

$$ \bigl\vert x^{\alpha } \bigr\vert \bigl\vert \Phi_{k}(x) \bigr\vert \lesssim \int_{b_{k}^{-1}\leq \vert \xi \vert \leq b_{k-2}^{-1}} \bigl\vert \partial^{\alpha } \lambda_{k} \bigl( \vert \xi \vert \bigr) \bigr\vert \,d\xi \leq C_{d}b_{k-2}^{-d}b_{k}^{d+1}. $$

This together with the fact $\vert x \vert ^{d+1}\leq C_{d} \sum_{\vert \beta \vert =d+1}\vert x^{\beta } \vert $ implies

3.12 $$ \bigl\vert \Phi_{k}(x) \bigr\vert \lesssim b_{k-2}^{-d}b_{k}^{d+1} \vert x \vert ^{-d-1}. $$

On the other hand, we have, for any multi-index *α* and any $j=1,2,\ldots ,d$,

$$ (2\pi i x)^{\alpha }\partial_{x_{j}}\Phi_{k}(x)= \int_{\mathbb{R}^{d}} \partial^{\alpha } \bigl(2\pi i \xi_{j}\lambda_{k} \bigl( \vert \xi \vert \bigr) \bigr)e^{2\pi ix\cdot \xi }\,d\xi . $$

Consequently,

$$ \bigl\vert x^{\alpha } \bigr\vert \bigl\vert \partial_{x_{j}} \Phi_{k}(x) \bigr\vert \lesssim \int_{b_{k}^{-1} \leq \vert \xi \vert \leq b_{k-2}^{-1}} \bigl\vert \partial^{\alpha } \bigl(2 \pi i \xi_{j}\lambda_{k} \bigl( \vert \xi \vert \bigr) \bigr) \bigr\vert \,d\xi . $$

From this inequality and the definition of $\lambda_{k}$, we have

$$ \vert x \vert ^{N} \bigl\vert \nabla \Phi_{k}(x) \bigr\vert \leq C_{N} \int_{b_{k}^{-1} \leq \vert \xi \vert \leq b_{k-2}^{-1}} \bigl(1+ \vert \xi \vert \bigr)^{-N+1} \,d\xi \leq C_{d,N}b_{k}^{N}b_{k-2}^{-d-1} \quad \forall N\in \mathbb{N}. $$

It follows that

$$ \bigl\vert b_{k}^{-1}x \bigr\vert ^{N} \bigl\vert \nabla \Phi_{k}(x) \bigr\vert \leq C_{d,N}b_{k-2}^{-d-1} \quad \forall N\in \mathbb{N}. $$

Consequently,

3.13 $$ \bigl\vert \nabla \Phi_{k}(x) \bigr\vert \leq C_{d}b_{k-2}^{-d-1} \bigl(1+ \bigl\vert b_{k}^{-1}x \bigr\vert \bigr)^{-d-1}. $$

By (3.12) and the fact that $\vert x-y \vert \geq {\vert x \vert }/{2}$ for any $\vert x \vert \geq 2\vert y \vert $,

3.14 $$\begin{aligned} \sum_{b_{k}\leq \vert y \vert } \int_{ \vert x \vert \geq 2 \vert y \vert } \bigl\vert \Phi_{k}(x-y)- \Phi_{k}(x) \bigr\vert \,dx&\leq \sum_{b_{k}\leq \vert y \vert }C_{d}b_{k-2}^{-d}b_{k}^{d+1} \int_{ \vert x \vert \geq 2 \vert y \vert } \vert x \vert ^{-d-1}\,dx \\ &\leq C_{d}\delta_{2}^{2d}\sum _{b_{k}\leq \vert y \vert }b_{k} \vert y \vert ^{-1} \\ &\leq C_{d,\delta_{2}}. \end{aligned}$$

Since $\vert x-\theta y \vert \geq {\vert x \vert }/{2}$ for any $\vert x \vert \geq 2\vert y \vert $ and $\theta \in [0,1]$, we see from (3.13), for any $\vert x \vert \geq 2\vert y \vert $, that there exists $\theta \in [0,1]$ such that

$$\begin{aligned} \bigl\vert \Phi_{k}(x-y)-\Phi_{k}(x) \bigr\vert & \lesssim \vert y \vert \bigl\vert \nabla \Phi_{k}(x-\theta y) \bigr\vert \\ &\leq C_{d} \vert y \vert b_{k-2}^{-d-1} \bigl(2+ \bigl\vert b_{k}^{-1}x \bigr\vert \bigr)^{-d-1}. \end{aligned}$$

This shows that

3.15 $$\begin{aligned} &\sum_{b_{k}> \vert y \vert } \int_{ \vert x \vert >2 \vert y \vert } \bigl\vert \Phi_{k}(x-y)- \Phi_{k}(x) \bigr\vert \,dx \\ &\quad = \sum_{b_{k}> \vert y \vert }C_{d}b_{k-2}^{-d-1} \vert y \vert \int_{ \vert x \vert >2 \vert y \vert } \bigl(2+ \bigl\vert b_{k} ^{-1}x \bigr\vert \bigr)^{-d-1}\,dx \\ &\quad \leq \sum_{b_{k}> \vert y \vert }C_{d}b_{k-2}^{-d-1} \vert y \vert b_{k}^{d} \int_{\mathbb{R}^{d}} \bigl(2+ \vert x \vert \bigr)^{-d-1}\,dx \\ &\quad \leq C_{d}\delta_{2}^{2(d+1)}\sum _{b_{k}> \vert y \vert }b_{k}^{-1} \vert y \vert \\ &\quad \leq C _{d,\delta_{2}}. \end{aligned}$$

Equation (3.15) together with (3.14) yields (3.11). Invoking [20, Theorem 4.6.1] we see that *T⃗* is bounded from $L^{r}(\mathbb{R}^{d})$ to $L^{r}(\ell^{2}, \mathbb{R}^{d})$ for any $1< r<\infty $. For any $1< r<\infty $, let $\mathcal{B}_{1}=L^{r}( \mathfrak{R}_{d})$ and $\mathcal{B}_{2}=L^{r}(\ell^{2},\mathfrak{R} _{d})$. By Fubini’s theorem and the $L^{r}(\mathbb{R}^{d})\rightarrow L^{r}(\ell^{2}, \mathbb{R}^{d})$ boundedness for *T⃗*,

3.16 $$\begin{array}{rl} {∥{∥\vec{T}(f_{\zeta })∥}_{B_{2}}∥}_{L^{r}(R^{d})} & ={\left(\int_{R^{d}}\int_{R_{d}}{\left(\sum_{k\in Z}|\Phi_{k}*f_{\zeta }(x){|}^{2}\right)}^{r/2}\;d\zeta \;dx\right)}^{1/r}\\ & ={\left(\int_{R_{d}}{∥\vec{T}f_{\zeta }∥}_{L^{r}(\ell^{2},R^{d})}^{r}\;d\zeta \right)}^{1/r}≲{\left(\int_{R_{d}}{∥f_{\zeta }∥}_{L^{r}(R^{d})}^{r}\;d\zeta \right)}^{1/r}\\ & \;≲{∥{∥f_{\zeta }∥}_{B_{1}}∥}_{L^{r}(R^{d})}. \end{array}$$

Note that

$$\begin{array}{rl} {∥\left(\vec{K}(x-y)-\vec{K}(x)\right)f_{\zeta }(x)∥}_{B_{2}} & ={\left(\int_{R_{d}}{\left(\sum_{k\in Z}|\left(\Phi_{k}(x-y)-\Phi_{k}(x)\right)f_{\zeta }(x){|}^{2}\right)}^{r/2}\;d\zeta \right)}^{1/r}\\ & ={\left(\sum_{k\in Z}|\Phi_{k}(x-y)-\Phi_{k}(x){|}^{2}\right)}^{1/2}{∥f_{\zeta }(x)∥}_{B_{1}}, \end{array}$$

which together with (3.11) implies

3.17 $$\underset{y\neq 0}{\sup}\int_{\left|x|\geq 2|y\right|}{∥\vec{K}(x-y)-\vec{K}(x)∥}_{B_{1}\to B_{2}}\;dx\leq C<\infty .$$

Applying (3.16)–(3.17) and Lemma 2.1 with $\rho (\cdot ) =\vert \cdot \vert $, we get (3.10).

We now define *J* by $J=G^{-1}(H^{-1}\otimes \delta_{ \mathbb{R}^{d-m}})$. Observe that *J* is a nonsingular linear transformation on $\mathbb{R}^{d}$. Denote $y=(y^{1}, y^{2})$, where $y^{1}=(y_{1},y_{2},\ldots ,y_{m})$ and $y^{2}=(y_{m+1}, y_{m+2}, \ldots ,y_{d})$. One can easily check that

3.18 $$ S_{k}f(x)= \vert J \vert \Phi_{k}\otimes \delta_{\mathbb{R}^{d-m}}*f^{J} \bigl(J^{t}x \bigr), $$

where $f^{J}(\xi )=\vert J \vert ^{-1}f((J^{t})^{-1}\xi)$. By the change of variables, (3.10) and (3.18),

$$\begin{array}{rl} & {∥{\left(\sum_{j\in Z}{∥{\left(\sum_{k\in Z}|S_{k}f_{j,\zeta }{|}^{2}\right)}^{1/2}∥}_{L^{r}(R_{d})}^{q}\right)}^{1/q}∥}_{L^{p}(R^{d})}^{p}\\ & \;\leq \int_{R^{d}}{\left(\sum_{j\in Z}{∥{\left(\sum_{k\in Z}||J|\Phi_{k}⊗\delta_{R^{d-m}}*f_{j,\zeta }^{J}\left(J^{t}x\right){|}^{2}\right)}^{1/2}∥}_{L^{r}(R_{d})}^{q}\right)}^{p/q}\;dx\\ & \;=|J{|}^{p-1}\int_{R^{d}}{\left(\sum_{j\in Z}{∥{\left(\sum_{k\in Z}|\Phi_{k}⊗\delta_{R^{d-m}}*f_{j,\zeta }^{J}(y){|}^{2}\right)}^{1/2}∥}_{L^{r}(R_{d})}^{q}\right)}^{p/q}\;dy\\ & \;=|J{|}^{p-1}\int_{R^{d-m}}\int_{R^{m}}{\left(\sum_{j\in Z}{∥{\left(\sum_{k\in Z}|\left[\Phi_{k}*f_{j,\zeta }^{J}\left(\cdot ,y^{2}\right)\right]\left(y^{1}\right){|}^{2}\right)}^{1/2}∥}_{L^{r}(R_{d})}^{q}\right)}^{p/q}\;dy^{1}\;dy^{2}\\ & \;≲|J{|}^{p-1}\int_{R^{d}}{\left(\sum_{j\in Z}{∥f_{j,\zeta }^{J}(y)∥}_{L^{r}(R_{d})}^{q}\right)}^{p/q}\;dy\\ & \;≲{∥{\left(\sum_{j\in Z}{∥f_{j,\zeta }∥}_{L^{r}(R_{d})}^{q}\right)}^{1/q}∥}_{L^{p}(R^{d})}^{p}. \end{array}$$

This completes the proof of Lemma 3.3. □

To prove Theorem 1.1, we need the following characterizations of the Triebel–Lizorkin spaces and Besov spaces.

### Lemma 3.4

([10])

*Let* $0<\alpha < \infty $ *and* *M* *be an integer such that* $M>\alpha $. *Let* $\triangle_{\zeta }^{M}f$ *be the* *Mth difference of* *f* *for an arbitrary function* *f* *defined on* $\mathbb{R}^{d}$.

(i) *If* $1< p<\infty $, $1< q\leq \infty $ *and* $1\leq r<\min \{p,q \}$, *then*
  $${∥f∥}_{{\dot{F}}_{\alpha ;r}^{p,q}(R^{d})}={∥{\left(\sum_{k\in Z}2^{kq\alpha }{\left(\int_{R_{d}}|{△}_{2^{-k}\zeta }^{M}f(\cdot ){|}^{r}\;d\zeta \right)}^{q/r}\right)}^{1/q}∥}_{L^{p}(R^{n})}$$
  *is an equivalent norm in* $\dot{F}_{\alpha }^{p,q}(\mathbb{R}^{n})$.
(ii) *If* $1\leq p<\infty $, $1\leq q\leq \infty $ *and* $1\leq r \leq p$, *then*
  $${∥f∥}_{{\dot{B}}_{\alpha ;r}^{p,q}(R^{d})}={\left(\sum_{k\in Z}2^{kq\alpha }{∥{\left(\int_{R_{d}}|{△}_{2^{-k}\zeta }^{M}f(\cdot ){|}^{r}\;d\zeta \right)}^{1/r}∥}_{L^{p}(R^{d})}^{q}\right)}^{1/q}$$
  *is an equivalent norm in* $\dot{B}_{\alpha }^{p,q}(\mathbb{R}^{d})$.

## Proof of Theorem 1.1

Let *h*, Ω, $\mathcal{R}_{\gamma }$ be given as in Theorem 1.1 and $\triangle_{\zeta }$ be the difference of *f*, i.e., $\triangle_{\zeta }f(x)= f(x+\zeta )-f(x)$. We split the proof of Theorem 1.1 in two parts.

*Step 1. Proof of* (ii) *of Theorem **1.1*. Let $\alpha \in (0,1)$, $\vert {1}/{p}-{1}/{2} \vert <\min \{{1}/{2},{1}/{\gamma '} \}$ and $q\in (1,\infty )$. Observe that

4.1 $$ \triangle_{\zeta } \bigl(\mathcal{M}_{h,\Omega ,\mathcal{P},\varphi }^{ \rho }f \bigr) (x)\leq \mathcal{M}_{h,\Omega ,\mathcal{P},\varphi }^{\rho }( \triangle_{\zeta }f) (x) \quad \forall x,\zeta \in \mathbb{R}^{d}. $$

By (4.1), Fubini’s theorem, Remark 1.1 and (ii) of Lemma 3.4, we have

$$\begin{array}{rl} & {∥M_{h,\Omega ,P,\varphi }^{\rho }f∥}_{{\dot{B}}_{\alpha }^{p,q}(R^{d})}\\ & \;≲{\left(\sum_{l\in Z}2^{lq\alpha }{∥{\left(\int_{R_{d}}|{△}_{2^{-l}\zeta }\left(M_{h,\Omega ,P,\varphi }^{\rho }f\right){|}^{p}\;d\zeta \right)}^{1/p}∥}_{L^{p}(R^{d})}^{q}\right)}^{1/q}\\ & \;≲{\left(\sum_{l\in Z}2^{lq\alpha }{\left(\int_{R_{d}}\int_{R^{d}}|M_{h,\Omega ,P,\varphi }^{\rho }({△}_{2^{-l}\zeta }f)(x){|}^{p}\;dx\;d\zeta \right)}^{q/p}\right)}^{1/q}\\ & \;≲{∥h∥}_{\Delta_{\gamma }(R_{+})}{∥\Omega ∥}_{H^{1}(S^{n-1})}{\left(\sum_{l\in Z}2^{lq\alpha }{\left(\int_{R^{d}}\int_{R_{d}}|{△}_{2^{-l}\zeta }f(x){|}^{p}\;d\zeta \;dx\right)}^{q/p}\right)}^{1/q}\\ & \;≲{∥h∥}_{\Delta_{\gamma }(R_{+})}{∥\Omega ∥}_{H^{1}(S^{n-1})}{∥f∥}_{{\dot{B}}_{\alpha }^{p,q}(R^{d})}. \end{array}$$

This proves the boundedness part of (ii) of Theorem 1.1. By (1.11), (4.1), Remark 1.1 and [17, Proposition 1], we can get the continuity part of (ii) of Theorem 1.1.

*Step 2. Proof of* (i) *of Theorem **1.1*. By (ii) of Theorem 1.1 and (1.6), we have

4.2 $${∥M_{h,\Omega ,P,\varphi }^{\rho }f∥}_{{\dot{F}}_{\alpha }^{p,q}(R^{d})}≲{∥h∥}_{\Delta_{\gamma }(R_{+})}{∥\Omega ∥}_{H^{1}(S^{n-1})}{∥f∥}_{{\dot{F}}_{\alpha }^{p,q}(R^{d})}$$

for $(1/p,1/q)\in \{(1/p,1/p):\vert {1}/{p}-{1}/{2} \vert <\min \{{1}/{2},{1}/ {\gamma '}\}\}$. Moreover, $\mathcal{M}_{h,\Omega ,\mathcal{P},\varphi }^{\rho }$ is continuous from $F_{\alpha }^{p,q}(\mathbb{R}^{d})$ to $\dot{F}_{\alpha }^{p,q}(\mathbb{R}^{d})$ for $(1/p, 1/q)\in \{(1/p,1/p):\vert {1}/{p}-{1}/{2} \vert <\min \{{1}/{2}, {1}/{\gamma '}\}\}$. Therefore, it suffices to prove (4.2) for $(1/p,1/q)\in \mathcal{R}_{\gamma }$ and $\mathcal{M}_{h,\Omega ,\mathcal{P},\varphi }^{\rho }$ is continuous from $F_{\alpha }^{p,q}(\mathbb{R}^{d})$ to $\dot{F}_{\alpha }^{p,q}( \mathbb{R}^{d})$ for $(1/p,1/q) \in \mathcal{R}_{\gamma }$.

By Lemma 3.1, to prove (4.2) for $(1/p,1/q)\in \mathcal{R}_{\gamma }$, it suffices to show that

4.3 $${∥M_{h,\Omega ,P,\varphi }^{\rho }f∥}_{{\dot{F}}_{\alpha }^{p,q}(R^{d})}≲{∥h∥}_{\Delta_{\gamma }(R_{+})}{∥f∥}_{{\dot{F}}_{\alpha }^{p,q}(R^{d})}$$

for $(1/p,1/q)\in \mathcal{R}_{\gamma }$ when Ω is a regular atom satisfying (3.1)–(3.3). Without loss of generality we may assume $\varepsilon =(0,\ldots ,0,1) \in \mathbb{R}^{n}$. We also only consider the case $0<\varrho <1/4$ and omit the easier case $\varrho \geq 1/4$. Let $M(m)$, $\{\Lambda_{\eta }\}_{\eta =1}^{M(m)}$, $\{\Gamma_{\eta } \}_{\eta =0}^{M(m)}$ and $\{L_{\eta }\}_{\eta =1}^{M(m)}$ be given as in [23]. Let $\sigma_{h,\Omega ,\Gamma ,t,\rho }$ be defined as in Sect. 3 and $\sigma_{k,t}^{\eta }= \sigma_{h, \Omega ,\Gamma_{\eta }(\varphi ),2^{k}t,\rho }$ with $\Gamma_{\eta }(\varphi )(x)=\Gamma_{\eta }(\varphi (\vert x \vert )x')$. For $\eta \in \{1,\ldots ,M(m)\}$, let $s(\eta )=\operatorname{rank} (L_{\eta })$. By [23, Lemma 6.1], there are two nonsingular linear transformations $H_{\eta }:\mathbb{R}^{s(\eta )}\rightarrow \mathbb{R}^{s(\eta )}$ and $G_{\eta }:\mathbb{R}^{d}\rightarrow \mathbb{R}^{d}$ such that

4.4 $$ \bigl\vert H_{\eta }\pi_{s(\eta )}^{d}G_{\eta }\xi \bigr\vert \leq \bigl\vert L_{\eta }(\xi ) \bigr\vert \leq \Lambda_{\eta } \bigl\vert H_{\eta }\pi_{s(\eta )}^{d}G_{\eta } \xi \bigr\vert \quad \forall \xi \in \mathbb{R}^{d}. $$

Let $\phi \in \mathcal{C}_{0}^{\infty }(\mathbb{R})$ such that $\phi \equiv 1$ for $\vert t \vert \leq 1/2$ and $\phi \equiv 0$ for $\vert t \vert >1$ and $\psi (t)=\phi (t^{2})$. Define the family of measures $\{\tau_{k,t} ^{\eta }\}_{t>0}$ by

4.5 $$ \widehat{\tau _{k,t}^{\eta }}(\xi )=\widehat{\sigma _{k,t}^{\eta }}( \xi )\Psi (\eta +1;k,t,\xi )-\widehat{\sigma _{k,t}^{\eta -1}}(\xi ) \Psi (\eta ;k,t,\xi ) $$

for $k\in \mathbb{Z}, t\in \mathbb{R}^{+}, \xi \in \mathbb{R}^{d}$ and $1\leq \eta \leq M(m)$, where $\{\delta (\eta )\}_{\eta =1}^{M(m)}$ and $\{l(\eta )\}_{\eta =1}^{M(m)}$ are given as in [23] and

$$ \Psi (\eta ;k,t,\xi )=\prod_{j=\eta }^{M(m)}\psi \bigl( \bigl\vert \varphi \bigl(2^{k-1} t \bigr)^{l(j)} \varrho^{\delta (j)}H_{j}\pi_{s(j)}^{d}G_{j} \xi \bigr\vert \bigr). $$

As in [34, (3.3)] we have

4.6 $$\begin{array}{rl} \left|\hat{\tau_{k,t}^{\eta }}(\xi )\right| & ≲{∥h∥}_{\Delta_{\gamma }(R_{+})}(\min\{1,\varphi {\left(2^{k-1}t\right)}^{l(\eta )}\varrho^{\delta (\eta )}\Lambda_{\eta }^{-1}|L_{\eta }(\xi )|,\\ & \;{\left(\varphi {\left(2^{k-1}t\right)}^{l(\eta )}\varrho^{\delta (\eta )}\Lambda_{\eta }^{-1}|L_{\eta }(\xi )|\right)}^{-1}\}{)}^{\gamma (\eta )} \end{array}$$

for $k\in \mathbb{Z}$, $t\in \mathbb{R}_{+}$, $\xi \in \mathbb{R}^{d}$ and $1\leq \eta \leq M(m)$, where $\{\gamma (\eta )\}_{\eta =1}^{M(m)}$ are given as in [23]. Let $B_{\varphi }$ be given as in Remark 1.2 and set $a_{k,\eta }=\varphi (2^{k})^{l(\eta )} \varrho^{\delta ( \eta )}\Lambda_{\eta }^{-1}$. We note that $B_{\varphi }^{l(\eta )} \leq \frac{a_{k+1,\eta }}{a_{k,\eta }} \leq c_{\varphi }^{l(\eta )}$ for any $k\in \mathbb{Z}$. This together with (4.6) shows that

4.7 $$\begin{array}{rl} & {\left(\int_{1}^{2}|\hat{\tau_{k,t}^{\eta }}(\xi ){|}^{2}\frac{dt}{t}\right)}^{1/2}\\ & \;≲{∥h∥}_{\Delta_{\gamma }(R_{+})}{\left(\min\left\{1,a_{k,\eta }|L_{\eta }(\xi )|,{\left(a_{k,\eta }|L_{\eta }(\xi )|\right)}^{-1}\right\}\right)}^{\gamma (\eta )} \end{array}$$

for any $k\in \mathbb{Z}$, $\xi \in \mathbb{R}^{d}$ and $1\leq \eta \leq M(m)$. By the argument similar to those used in deriving [34, (3.9)], we obtain

4.8 $$ \mathcal{M}_{h,\Omega ,\mathcal{P},\varphi }^{\rho }f(x) \leq C_{ \rho }\sum _{\eta =1}^{M(m)} \biggl(\sum_{k\in \mathbb{Z}} \int_{1}^{2} \bigl\vert \tau_{k,t}^{\eta }*f(x) \bigr\vert ^{2}\frac{dt}{t} \biggr)^{1/2}. $$

Equation (4.8) together with (4.1), (i) of Lemma 3.4 and Minkowski’s inequality implies

4.9 $$\begin{array}{rl} & {∥M_{h,\Omega ,P,\varphi }^{\rho }f∥}_{{\dot{F}}_{\alpha }^{p,q}(R^{d})}\\ & \;≲{∥{\left(\sum_{l\in Z}2^{lq\alpha }{\left(\int_{R_{d}}|{△}_{2^{-l}\zeta }\left(M_{h,\Omega ,P,\varphi }^{\rho }f\right)|\;d\zeta \right)}^{q}\right)}^{1/q}∥}_{L^{p}(R^{d})}\\ & \;≲{∥{\left(\sum_{l\in Z}2^{lq\alpha }{\left(\int_{R_{d}}|M_{h,\Omega ,P,\varphi }^{\rho }({△}_{2^{-l}\zeta }f)|\;d\zeta \right)}^{q}\right)}^{1/q}∥}_{L^{p}(R^{d})}\\ & \;≲\sum_{\eta =1}^{M(m)}{∥{\left(\sum_{l\in Z}2^{lq\alpha }{\left(\int_{R_{d}}{\left(\sum_{k\in Z}\int_{1}^{2}|\tau_{k,t}^{\eta }*{△}_{2^{-l}\zeta }f{|}^{2}\frac{dt}{t}\right)}^{1/2}\;d\zeta \right)}^{q}\right)}^{1/q}∥}_{L^{p}(R^{d})} \end{array}$$

for $0<\alpha <1$ and $1< p$, $q<\infty $. Thus, to prove (4.3), it suffices to show that

4.10 $$\begin{array}{rl} & {∥{\left(\sum_{l\in Z}2^{lq\alpha }{\left(\int_{R_{d}}{\left(\sum_{k\in Z}\int_{1}^{2}|\tau_{k,t}^{\eta }*{△}_{2^{-l}\zeta }f{|}^{2}\frac{dt}{t}\right)}^{1/2}\;d\zeta \right)}^{q}\right)}^{1/q}∥}_{L^{p}(R^{d})}\\ & \;≲{∥h∥}_{\Delta_{\gamma }(R_{+})}{∥f∥}_{{\dot{F}}_{\alpha }^{p,q}(R^{d})} \end{array}$$

for any $1\leq \eta \leq M(m)$, $\alpha \in (0,1)$ and $(1/p,1/q) \in \mathcal{R}_{\gamma }$.

We now prove (4.10). Let $\{\upsilon_{k,\eta }\}_{k\in \mathbb{Z}}$ be a collection of $\mathcal{C}^{\infty }(\mathbb{R}_{+})$ with the following properties:

$$ \operatorname{supp} (\upsilon_{k,\eta })\subset \bigl[a_{k+1,\eta }^{-1},a_{k-1, \eta }^{-1} \bigr]; \qquad 0\leq \upsilon_{k,\eta }(t)\leq 1;\qquad \sum _{k\in \mathbb{Z}}\upsilon_{k,\eta }(t)=1. $$

Define the sequence of multiplier operators $\{\Upsilon_{k,\eta }\} _{k\in \mathbb{Z}}$ on $\mathbb{R}^{d}$ by

$$ \widehat{\Upsilon _{k,\eta }f}(\xi )=\upsilon_{k,\eta } \bigl( \bigl\vert H_{\eta } \pi_{s(\eta )}^{d}G_{\eta }\xi \bigr\vert \bigr)\hat{f}(\xi ). $$

By Minkowski’s inequality,

4.11 $$\begin{array}{rl} & {∥{\left(\sum_{l\in Z}2^{lq\alpha }{\left(\int_{R_{d}}{\left(\sum_{k\in Z}\int_{1}^{2}|\tau_{k,t}^{\eta }*{△}_{2^{-l}\zeta }f{|}^{2}\frac{dt}{t}\right)}^{1/2}\;d\zeta \right)}^{q}\right)}^{1/q}∥}_{L^{p}(R^{d})}\\ & \;={∥{\left(\sum_{l\in Z}2^{lq\alpha }{\left(\int_{R_{d}}{\left(\sum_{k\in Z}\int_{1}^{2}|\tau_{k,t}^{\eta }*\sum_{j\in Z}{ϒ}_{j+k,\eta }{△}_{2^{-l}\zeta }f{|}^{2}\frac{dt}{t}\right)}^{1/2}\;d\zeta \right)}^{q}\right)}^{1/q}∥}_{L^{p}(R^{d})}\\ & \;\leq \sum_{j\in Z}{∥{\left(\sum_{l\in Z}2^{lq\alpha }{\left(\int_{R_{d}}{\left(\sum_{k\in Z}\int_{1}^{2}|\tau_{k,t}^{\eta }*{ϒ}_{j+k,\eta }{△}_{2^{-l}\zeta }f{|}^{2}\frac{dt}{t}\right)}^{1/2}\;d\zeta \right)}^{q}\right)}^{1/q}∥}_{L^{p}(R^{d})}. \end{array}$$

Define the mixed norm ${∥\cdot ∥}_{E_{\alpha }^{p,q}}$ for measurable functions on $\mathbb{R}^{d}\times \mathfrak{R}_{d} \times \mathbb{Z} \times \mathbb{Z}\times \mathbb{R}_{+}$ by

$${∥g∥}_{E_{\alpha }^{p,q}}:={∥{\left(\sum_{l\in Z}2^{lq\alpha }{\left(\int_{R_{d}}{\left(\sum_{k\in Z}\int_{R_{+}}|g(x,t,\zeta ,l,k){|}^{2}\frac{dt}{t}\right)}^{1/2}\;d\zeta \right)}^{q}\right)}^{1/q}∥}_{L^{p}(R^{d})}.$$

For any $j\in \mathbb{Z}$, let

$$ V_{j,\eta }(f) (x,t,\zeta ,l,k):=\tau_{k,t}^{\eta }* \Upsilon_{j+k, \eta }\triangle_{2^{-l}\zeta }f(x)\chi_{[1,2]}(t). $$

Then (4.11) reduces to the following:

4.12 $$\begin{array}{rl} & {∥{\left(\sum_{l\in Z}2^{lq\alpha }{\left(\int_{R_{d}}{\left(\sum_{k\in Z}\int_{1}^{2}|\tau_{k,t}^{\eta }*{△}_{2^{-l}\zeta }f{|}^{2}\frac{dt}{t}\right)}^{1/2}\;d\zeta \right)}^{q}\right)}^{1/q}∥}_{L^{p}(R^{d})}\\ & \;\leq \sum_{j\in Z}{∥V_{j,\eta }(f)∥}_{E_{\alpha }^{p,q}}. \end{array}$$

Thus, to prove (4.10), it suffices to show that for any $\alpha \in (0,1)$ and $(1/p,1/q)\in \mathcal{R}_{\gamma }$, there exists $\delta >0$ such that

4.13 $$\begin{array}{rl} & {∥V_{j,\eta }(f)∥}_{E_{\alpha }^{p,q}}\\ & \;≲{∥h∥}_{\Delta_{\gamma }(R_{+})}B_{\varphi }^{-\delta |j|}{∥f∥}_{{\dot{F}}_{\alpha }^{p,q}(R^{d})}. \end{array}$$

By (4.7), Hölder’s inequality, Minkowski’s inequality, Fubini’s theorem, Plancherel’s theorem and (ii) of Lemma 3.4,

$$\begin{array}{rl} & {∥V_{j,\eta }(f)∥}_{E_{\alpha }^{2,2}}^{2}\\ & \;=\int_{R^{d}}\sum_{l\in Z}2^{2l\alpha }{\left(\int_{R_{d}}{\left(\sum_{k\in Z}\int_{1}^{2}|\tau_{k,t}^{\eta }*{ϒ}_{j+k,\eta }{△}_{2^{-l}\zeta }f(x){|}^{2}\frac{dt}{t}\right)}^{1/2}\;d\zeta \right)}^{2}\;dx\\ & \;≲\sum_{l\in Z}2^{2l\alpha }\int_{R_{d}}\int_{1}^{2}\sum_{k\in Z}\int_{R^{d}}|\tau_{k,t}^{\eta }*{ϒ}_{j+k,\eta }{△}_{2^{-l}\zeta }f(x){|}^{2}\;dx\frac{dt}{t}\;d\zeta \\ & \;≲\sum_{l\in Z}2^{2l\alpha }\int_{R_{d}}\sum_{k\in Z}\int_{a_{j+k+1,\eta }^{-1}\leq |L_{\eta }(\xi )|\leq \Lambda_{\eta }a_{j+k-1,\eta }^{-1}}\int_{1}^{2}|\hat{\tau_{k,t}^{\eta }}(x){|}^{2}\frac{dt}{t}|\hat{{△}_{2^{-l}\zeta }f}(x){|}^{2}\;dx\;d\zeta \\ & \;≲{∥h∥}_{\Delta_{\gamma }(R_{+})}B_{\varphi }^{-2\delta l(\eta )|j|}{∥f∥}_{{\dot{B}}_{\alpha }^{2,2}(R^{d})}^{2}. \end{array}$$

Combining this inequality with (1.4) implies that

4.14 $${∥V_{j,\eta }(f)∥}_{E_{\alpha }^{2,2}}≲{∥h∥}_{\Delta_{\gamma }(R_{+})}B_{\varphi }^{-2\delta l(\eta )|j|}{∥f∥}_{{\dot{F}}_{\alpha }^{2,2}(R^{d})}.$$

Thus, we shall prove

4.15 $${∥V_{j,\eta }(f)∥}_{E_{\alpha }^{p,q}}≲{∥h∥}_{\Delta_{\gamma }(R_{+})}{∥f∥}_{{\dot{F}}_{\alpha }^{p,q}(R^{d})}$$

for any $\alpha \in (0,1)$ and $(1/p,1/q)\in \mathcal{R}_{\gamma }$. Indeed, (4.13) follows easily from the interpolation between (4.14) and (4.15).

For $1\leq \iota \leq M(m)$, let $\Phi^{\iota }$ be a radial function in $\mathcal{S}(\mathbb{R}^{s(\iota )})$. Define $J_{\iota }$ and $X_{\iota }$ by

$$ J_{\iota }f(x):=f \bigl(G_{\iota }^{t} \bigl(H_{\iota }^{t}\otimes id_{\mathbb{R} ^{d-s(\iota )}} \bigr)x \bigr) \quad \mbox{and} \quad X_{\iota }f(x)=\sup_{k\in \mathbb{Z}}\sup _{t\in [1,2]} \bigl\vert X_{k,t;\iota }f(x) \bigr\vert $$

where

$$ X_{k,t;\iota }f(x)=J_{\iota }^{-1} \bigl(( \Phi_{k,t;\iota } \otimes \delta_{\mathbb{R}^{d-s(\iota )}})*J_{\iota }f \bigr) (x), $$

and

$$ \Phi_{k,t;\iota } \bigl(x^{0} \bigr)= \bigl(\varphi \bigl(2^{k-1}t \bigr)^{l(\iota )} \varrho^{\delta (\iota )} \bigr)^{-s(\iota )} \Phi^{\iota } \bigl(\varphi \bigl(2^{k-1}t \bigr)^{-l( \iota )}\varrho^{-\delta (\iota )}x^{0} \bigr),\quad x^{0}\in \mathbb{R}^{s( \iota )}. $$

One can easily check that

4.16 $$ \bigl\vert X_{\iota }f(x) \bigr\vert \leq C_{\iota } \bigl[J_{\iota }^{-1}\circ (\mathrm{M}_{(s( \iota ))}\otimes id_{\mathbb{R}^{d-s(\iota )}})\circ J_{\iota } \bigr](f) (x). $$

Inequation (4.16) together with Lemma 2.2 shows that

4.17 $$\begin{array}{rl} & {∥{\left(\sum_{j\in Z}{∥{\left(\sum_{k\in Z}|X_{\iota }g_{k,j,\zeta }{|}^{2}\right)}^{1/2}∥}_{L^{r}(R_{d})}^{q}\right)}^{1/q}∥}_{L^{p}(R^{d})}\\ & \;≲{∥{\left(\sum_{j\in Z}{∥{\left(\sum_{k\in Z}|g_{k,j,\zeta }{|}^{2}\right)}^{1/2}∥}_{L^{r}(R_{d})}^{q}\right)}^{1/q}∥}_{L^{p}(R^{d})} \end{array}$$

for any $1\leq \iota \leq M(m)$ and $1< p$, $q, r<\infty $. For any $1\leq \eta \leq M(m)$, we define $X^{\eta }f= X_{\eta }\circ X_{ \eta +1}\circ \cdots \circ X_{M(m)}f$. Then (4.17) shows that

4.18 $$\begin{array}{rl} & {∥{\left(\sum_{j\in Z}{∥{\left(\sum_{k\in Z}|X^{\iota }g_{k,j,\zeta }{|}^{2}\right)}^{1/2}∥}_{L^{r}(R_{d})}^{q}\right)}^{1/q}∥}_{L^{p}(R^{d})}\\ & \;≲{∥{\left(\sum_{j\in Z}{∥{\left(\sum_{k\in Z}|g_{k,j,\zeta }{|}^{2}\right)}^{1/2}∥}_{L^{r}(R_{d})}^{q}\right)}^{1/q}∥}_{L^{p}(R^{d})} \end{array}$$

for any $1\leq \iota \leq M(m)$ and $1< p,q,r<\infty $. On the other hand, we get from (4.5) that

$$\begin{aligned} \tau_{k,t}^{\eta }*f&=\sigma_{k,t}^{\eta }*(X_{k,t;\eta +1} \circ X_{k,t; \eta +2}\circ \cdots \circ X_{k,t;M(m)}f) \\ &\quad {} - \sigma_{k,t}^{\eta -1}*(X _{k,t;\eta }\circ X_{k,t;\eta +1} \circ \cdots \circ X_{k,t;M(m)}f). \end{aligned}$$

It follows that

4.19 $$\begin{aligned}& \int_{1}^{2} \bigl\vert \tau_{k,t}^{1}*f(x) \bigr\vert ^{2}\frac{dt}{t} \leq \int_{1}^{2} \bigl\vert \bigl\vert \sigma_{k,t}^{1} \bigr\vert *X^{\eta +1}f(x) \bigr\vert ^{2}\frac{dt}{t}; \end{aligned}$$

4.20 $$\begin{aligned}& \begin{aligned} \int_{1}^{2} \bigl\vert \tau_{k,t}^{\eta }*f(x) \bigr\vert ^{2}\frac{dt}{t} & \leq 2 \biggl( \int _{1}^{2} \bigl\vert \bigl\vert \sigma_{k,t}^{\eta } \bigr\vert *X^{\eta +1}f(x) \bigr\vert ^{2} \frac{dt}{t} \\ &\quad {}+ \int_{1}^{2} \bigl\vert \bigl\vert \sigma_{k,t}^{\eta -1}\bigl\vert *X^{\eta }f(x) \bigr\vert ^{2}\frac{dt}{t} \biggr)\quad \mbox{for } 2\leq \eta \leq M(m). \end{aligned} \end{aligned}$$

Combining (4.18)–(4.20) with Lemma 3.2 shows that

4.21 $$\begin{array}{rl} & {∥{\left(\sum_{l\in Z}{\left(\int_{R_{d}}{\left(\sum_{k\in Z}\int_{1}^{2}|\tau_{k,t}^{\eta }*g_{l,\zeta ,k}{|}^{2}\frac{dt}{t}\right)}^{1/2}\;d\zeta \right)}^{q}\right)}^{1/q}∥}_{L^{p}(R^{d})}\\ & \;≲{∥h∥}_{\Delta_{\gamma }(R_{+})}{∥{\left(\sum_{l\in Z}{∥{\left(\sum_{k\in Z}|g_{l,\zeta ,k}{|}^{2}\right)}^{1/2}∥}_{L^{r}(R_{d})}^{q}\right)}^{1/q}∥}_{L^{p}(R^{d})} \end{array}$$

for $\{g_{l,\zeta ,k}\}\in L^{p}(\ell^{q}(L^{r}(\ell^{2}, \mathfrak{R}_{d})),\mathbb{R}^{d})$ with $(1/p,1/q,1/r)$ belonging to the interior of the convex hull of three cubes $(\frac{1}{2}, \frac{1}{2}+\frac{1}{\max \{2, \gamma '\}})^{3}$, $(\frac{1}{2}-\frac{1}{ \max \{2,\gamma '\}}, \frac{1}{2})^{3}$, and $(\frac{1}{2\gamma }, 1-\frac{1}{2 \gamma })^{3}$. Let $\alpha \in (0,1)$ and $(1/p,1/q)\in \mathcal{R} _{\gamma }$. We can choose $1< r<\min \{p,q\}$ such that $(1/p,1/q,1/r)$ belongs to the interior of the convex hull of three cubes $( \frac{1}{2},\frac{1}{2}+ \frac{1}{\max \{2,\gamma '\}})^{3}$, $(\frac{1}{2} -\frac{1}{\max \{2,\gamma '\}},\frac{1}{2})^{3}$, and $(\frac{1}{2\gamma },1-\frac{1}{2\gamma })^{3}$. By (4.21) and Lemmas 3.3 and 3.4, we obtain

$$\begin{array}{rl} {∥V_{j,\eta }(f)∥}_{E_{\alpha }^{p,q}} & ≲{∥h∥}_{\Delta_{\gamma }(R_{+})}{∥{\left(\sum_{l\in Z}2^{lq\alpha }{∥{\left(\sum_{k\in Z}|{ϒ}_{j+k,\eta }{△}_{2^{-l}\zeta }f{|}^{2}\right)}^{1/2}∥}_{L^{r}(R_{d})}^{q}\right)}^{1/q}∥}_{L^{p}(R^{d})}\\ & ≲{∥h∥}_{\Delta_{\gamma }(R_{+})}{∥{\left(\sum_{l\in Z}2^{lq\alpha }{∥{△}_{2^{-l}\zeta }f∥}_{L^{r}(R_{d})}^{q}\right)}^{1/q}∥}_{L^{p}(R^{d})}\\ & ≲{∥h∥}_{\Delta_{\gamma }(R_{+})}{∥f∥}_{{\dot{F}}_{\alpha }^{p,q}(R^{d})}. \end{array}$$

This yields (4.15) and completes the proof of the boundedness part of (i).

We now prove the continuity part of (i). By Lemma 3.1, Minkowski’s inequality and (4.9)–(4.10), we have

4.22 $$\begin{array}{rl} & {∥{\left(\sum_{l\in Z}2^{lq\alpha }{\left(\int_{R_{d}}|M_{h,\Omega ,P,\varphi }^{\rho }({△}_{2^{-l}\zeta }f)|\;d\zeta \right)}^{q}\right)}^{1/q}∥}_{L^{p}(R^{d})}\\ & \;≲\sum_{j}|c_{j}|{∥{\left(\sum_{l\in Z}2^{lq\alpha }{\left(\int_{R_{d}}|M_{h,\Omega_{j},P,\varphi }^{\rho }({△}_{2^{-l}\zeta }f)|\;d\zeta \right)}^{q}\right)}^{1/q}∥}_{L^{p}(R^{d})}\\ & \;≲{∥\Omega ∥}_{H^{1}(S^{n-1})}{∥f∥}_{{\dot{F}}_{\alpha }^{p,q}(R^{d})} \end{array}$$

for $\alpha \in (0,1)$ and $(1/p,1/q)\in \mathcal{R}_{\gamma }$. Let $\alpha \in (0,1)$, $(1/p,1/q)\in \mathcal{R}_{\gamma }$ and $f_{j}\rightarrow f$ in $F_{\alpha }^{p,q}(\mathbb{R}^{d})$ as $j\rightarrow \infty $. We want to show that $\mathcal{M}_{h,\Omega , \mathcal{P},\varphi }^{\rho }f_{j} \rightarrow \mathcal{M}_{h,\Omega ,\mathcal{P}, \varphi }^{\rho }f$ in $\dot{F}_{\alpha }^{p,q}( \mathbb{R}^{d})$ as $j\rightarrow \infty $. We shall prove this claim by contradiction. Without loss of generality we may assume that there exists $c>0$ such that

$${∥M_{h,\Omega ,P,\varphi }^{\rho }f_{j}-M_{h,\Omega ,P,\varphi }^{\rho }f∥}_{{\dot{F}}_{\alpha }^{p,q}(R^{d})}>c$$

for every *j*.

By (1.5) we see that $f_{j}\rightarrow f$ in $\dot{F}_{\alpha }^{p,q}( \mathbb{R}^{d})$ and in $L^{p}(\mathbb{R}^{d})$ as $j\rightarrow \infty $. It follows from (1.11) that $\mathcal{M}_{h,\Omega , \mathcal{P},\varphi }^{\rho }f_{j}\rightarrow \mathcal{M}_{h,\Omega , \mathcal{P},\varphi }^{\rho }f$ in $L^{p}(\mathbb{R}^{d})$ as $j\rightarrow \infty $. Then by extracting a subsequence we may assume that $\vert \mathcal{M}_{h,\Omega ,\mathcal{P},\varphi }^{\rho } f_{j}(x)-\mathcal{M}_{h,\Omega ,\mathcal{P}, \varphi }^{\rho }f(x) \vert \rightarrow 0$ as $j\rightarrow \infty $ for almost every $x\in \mathbb{R}^{d}$. It follows that $\triangle_{2^{-l}\zeta }(\mathcal{M}_{h,\Omega , \mathcal{P}, \varphi }^{\rho }f_{j}-\mathcal{M}_{h,\Omega , \mathcal{P}, \varphi }^{\rho }f)(x)\rightarrow 0$ as $j\rightarrow \infty $ for every $(l,\zeta )\in \mathbb{Z}\times \mathfrak{R}_{d}$ and almost every $x\in \mathbb{R}^{d}$. We get from (4.1) and (1.11) that

$$\begin{aligned}& \bigl\vert \triangle_{2^{-l}\zeta } \bigl(\mathcal{M}_{h,\Omega ,\mathcal{P},\varphi }^{\rho }f_{j}- \mathcal{M}_{h,\Omega ,\mathcal{P},\varphi }^{\rho }f \bigr) (x) \bigr\vert \\& \quad \leq 2 \mathcal{M}_{h,\Omega ,\mathcal{P},\varphi }^{\rho }( \triangle_{2^{-l}\zeta }f) (x)+ \mathcal{M}_{h,\Omega ,\mathcal{P}, \varphi }^{\rho } \bigl(\triangle_{2^{-l}\zeta }(f_{j}-f) \bigr) (x) \end{aligned}$$

for $(x,l,\zeta )\in \mathbb{R}^{d}\times \mathbb{Z}\times \mathfrak{R}_{d}$. For convenience, we set

$${∥g∥}_{p,q,\alpha }:={∥{\left(\sum_{l\in Z}2^{lq\alpha }{\left(\int_{R_{d}}|g(x,l,\zeta )|\;d\zeta \right)}^{q}\right)}^{1/q}∥}_{L^{p}(R^{d})}$$

for $\alpha \in \mathbb{R}$ and $(p,q)\in (1,\infty )^{2}$. It follows from (i) of Lemma 3.4 that ${∥f∥}_{{\dot{F}}_{\alpha }^{p,q}(R^{d})}∼{∥{△}_{2^{-l}\zeta }f∥}_{p,q,\alpha }$ for $\alpha \in (0,1)$ and $(p,q)\in (1,\infty )^{2}$. By (4.22) we obtain

$$\begin{array}{rl} {∥M_{h,\Omega ,P,\varphi }^{\rho }({△}_{2^{-l}\zeta }f)∥}_{p,q,\alpha } & ≲{∥{\left(\sum_{l\in Z}2^{lq\alpha }{\left(\int_{R_{d}}|M_{h,\Omega ,P,\varphi }^{\rho }({△}_{2^{-l}\zeta }f)|\;d\zeta \right)}^{q}\right)}^{1/q}∥}_{L^{p}(R^{d})}\\ & ≲{∥f∥}_{{\dot{F}}_{\alpha }^{p,q}(R^{d})}. \end{array}$$

It follows that ${∥M_{h,\Omega ,P,\varphi }^{\rho }({△}_{2^{-l}\zeta }(f_{j}-f))∥}_{p,q,\alpha }≲{∥f_{j}-f∥}_{{\dot{F}}_{\alpha }^{p,q}(R^{d})}\to 0$ as $j\rightarrow \infty $. One can extract a subsequence such that $\sum_{j=1}^{\infty }{∥M_{h,\Omega ,P,\varphi }^{\rho }({△}_{2^{-l}\zeta }(f_{j}-f))∥}_{p,q,\alpha }<\infty$. Define a function $G:\mathbb{R}^{d}\times \mathbb{Z}\times \mathfrak{R}_{d} \rightarrow \mathbb{R}$ by

$$ G(x,l,\zeta )=\sum_{j=1}^{\infty } \mathcal{M}_{h,\Omega , \mathcal{P}, \varphi }^{\rho } \bigl(\triangle_{2^{-l}\zeta }(f_{j}-f) \bigr) (x)+2\mathcal{M} _{h,\Omega ,\mathcal{P},\varphi }^{\rho }(\triangle_{2^{-l}\zeta }f) (x). $$

One can easily check that ${∥G∥}_{p,q,\alpha }<\infty$ and

4.23 $$\begin{aligned} & \bigl\vert \triangle_{2^{-l}\zeta } \bigl(\mathcal{M}_{h,\Omega ,\mathcal{P},\varphi }^{\rho }f_{j}- \mathcal{M}_{h,\Omega ,\mathcal{P},\varphi }^{\rho }f \bigr) (x) \bigr\vert \\ &\quad \leq G(x,l,\zeta )\quad \mbox{for almost every } (x,l,\zeta )\in \mathbb{R}^{d}\times \mathbb{Z}\times \mathfrak{R}_{d}. \end{aligned}$$

Since ${∥G∥}_{p,q,\alpha }<\infty$, we have $\int_{\mathfrak{R}_{d}}G(x,k, \zeta )\,d\zeta <\infty $ for every $k\in \mathbb{Z}$ and almost every $x\in \mathbb{R}^{d}$. Inequation (4.23) together with the dominated convergence theorem leads to

4.24 $$ \int_{\mathfrak{R}_{d}} \bigl\vert \triangle_{2^{-l}\zeta } \bigl( \mathcal{M}_{h, \Omega ,\mathcal{P},\varphi }^{\rho }f_{j}-\mathcal{M}_{h,\Omega , \mathcal{P},\varphi }^{\rho }f \bigr) (x) \bigr\vert \,d\zeta \rightarrow 0 \quad \mbox{as } j \rightarrow \infty $$

for every $l\in \mathbb{Z}$ and almost every $x\in \mathbb{R}^{d}$. By the fact ${∥G∥}_{p,q,\alpha }<\infty$ again,

4.25 $$ \biggl(\sum_{l\in \mathbb{Z}}2^{lq\alpha } \biggl( \int_{\mathfrak{R}_{d}}G(x,l, \zeta )\,d\zeta \biggr)^{q} \biggr)^{1/q}< \infty $$

for almost every $x\in \mathbb{R}^{d}$. Using (4.23) we obtain

4.26 $$ \int_{\mathfrak{R}_{d}} \bigl\vert \triangle_{2^{-l}\zeta } \bigl( \mathcal{M}_{h, \Omega ,\mathcal{P},\varphi }^{\rho }f_{j}-\mathcal{M}_{h,\Omega , \mathcal{P},\varphi }^{\rho }f \bigr) (x) \bigr\vert \,d\zeta \leq \int_{\mathfrak{R}_{d}}G(x,l, \zeta )\,d\zeta $$

for almost every $x\in \mathbb{R}^{d}$ and $l\in \mathbb{Z}$. It follows from (4.24)–(4.26) and the dominated convergence theorem that

4.27 $$ \biggl(\sum_{l\in \mathbb{Z}}2^{lq\alpha } \biggl( \int_{\mathfrak{R}_{d}} \bigl\vert \triangle_{2^{-l}\zeta } \bigl( \mathcal{M}_{h,\Omega ,\mathcal{P},\varphi } ^{\rho }f_{j}-\mathcal{M}_{h,\Omega ,\mathcal{P},\varphi }^{\rho }f \bigr) (x) \bigr\vert \,d\zeta \biggr)^{q} \biggr)^{1/q} \rightarrow 0\quad \mbox{as } j\rightarrow \infty $$

for almost every $x\in \mathbb{R}^{d}$. By (4.23) again, we have

4.28 $$\begin{aligned} & \biggl(\sum_{l\in \mathbb{Z}}2^{lq\alpha } \biggl( \int_{\mathfrak{R}_{d}} \bigl\vert \triangle_{2^{-l}\zeta } \bigl( \mathcal{M}_{h,\Omega ,\mathcal{P},\varphi } ^{\rho }f_{j}-\mathcal{M}_{h,\Omega ,\mathcal{P},\varphi }^{\rho }f \bigr) (x) \bigr\vert \,d\zeta \biggr)^{q} \biggr)^{1/q} \\ &\quad \leq \biggl(\sum_{l\in \mathbb{Z}}2^{lq \alpha } \biggl( \int_{\mathfrak{R}_{d}} \bigl\vert G(x,l,\zeta ) \bigr\vert \,d\zeta \biggr)^{q} \biggr)^{1/q} \end{aligned}$$

for almost every $x\in \mathbb{R}^{d}$. By (4.27)–(4.28), the fact ${∥G∥}_{p,q,\alpha }<\infty$ and the dominated convergence theorem, we get

$${∥{△}_{2^{-l}\zeta }\left(M_{h,\Omega ,P,\varphi }^{\rho }f_{j}-M_{h,\Omega ,P,\varphi }^{\rho }f\right)∥}_{p,q,\alpha }\to 0\;\text{as }j\to \infty .$$

This leads to ${∥M_{h,\Omega ,P,\varphi }^{\rho }f_{j}-M_{h,\Omega ,P,\varphi }^{\rho }f∥}_{{\dot{F}}_{\alpha }^{p,q}(R^{d})}\to 0$ as $j\rightarrow \infty $, which is a contradiction. □
